# Supplementary figures and images for: Saccharomyces cerevisiae Rev7 promotes non-homologous end-joining by blocking Mre11 nuclease and Rad50’s ATPase activities and homologous recombination
Source: eLife. 2024 Dec 4;13:RP96933. doi: 10.7554/eLife.96933 (PMC11616998; doi:10.7554/eLife.96933)

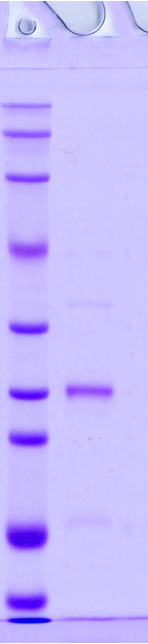

Supplement: Figure 1—figure supplement 1—source data 1. [file elife-96933-fig1-figsupp1-data1.zip › Figure 1-figure supplement 1-source data 1/Figure 1-figure supplement 1E.tif]

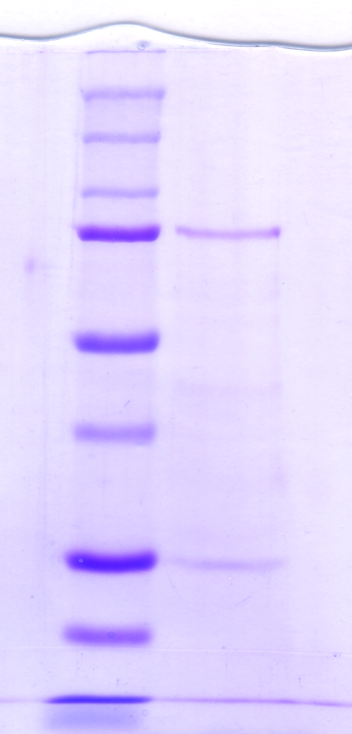

Supplement: Figure 1—figure supplement 1—source data 1. [file elife-96933-fig1-figsupp1-data1.zip › Figure 1-figure supplement 1-source data 1/Figure 1-figure supplement 1D.tif]

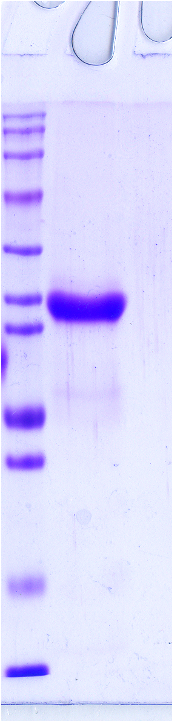

Supplement: Figure 1—figure supplement 1—source data 1. [file elife-96933-fig1-figsupp1-data1.zip › Figure 1-figure supplement 1-source data 1/Figure 1-figure supplement 1F.tif]

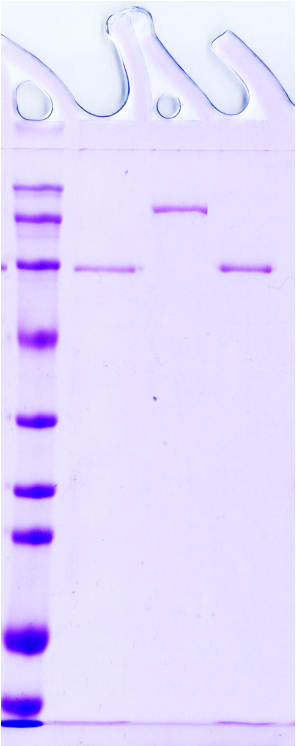

Supplement: Figure 1—figure supplement 1—source data 1. [file elife-96933-fig1-figsupp1-data1.zip › Figure 1-figure supplement 1-source data 1/Figure 1-figure supplement 1C.tif]

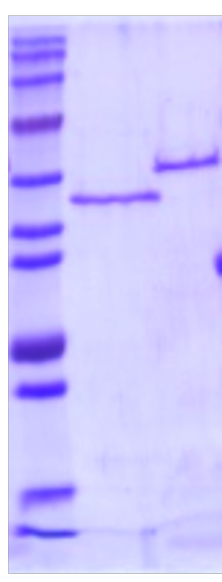

Supplement: Figure 1—figure supplement 1—source data 1. [file elife-96933-fig1-figsupp1-data1.zip › Figure 1-figure supplement 1-source data 1/Figure 1-figure supplement 1B.tif]

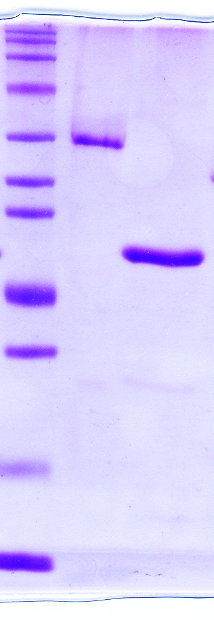

Supplement: Figure 1—figure supplement 1—source data 1. [file elife-96933-fig1-figsupp1-data1.zip › Figure 1-figure supplement 1-source data 1/Figure 1-figure supplement 1A.tif]

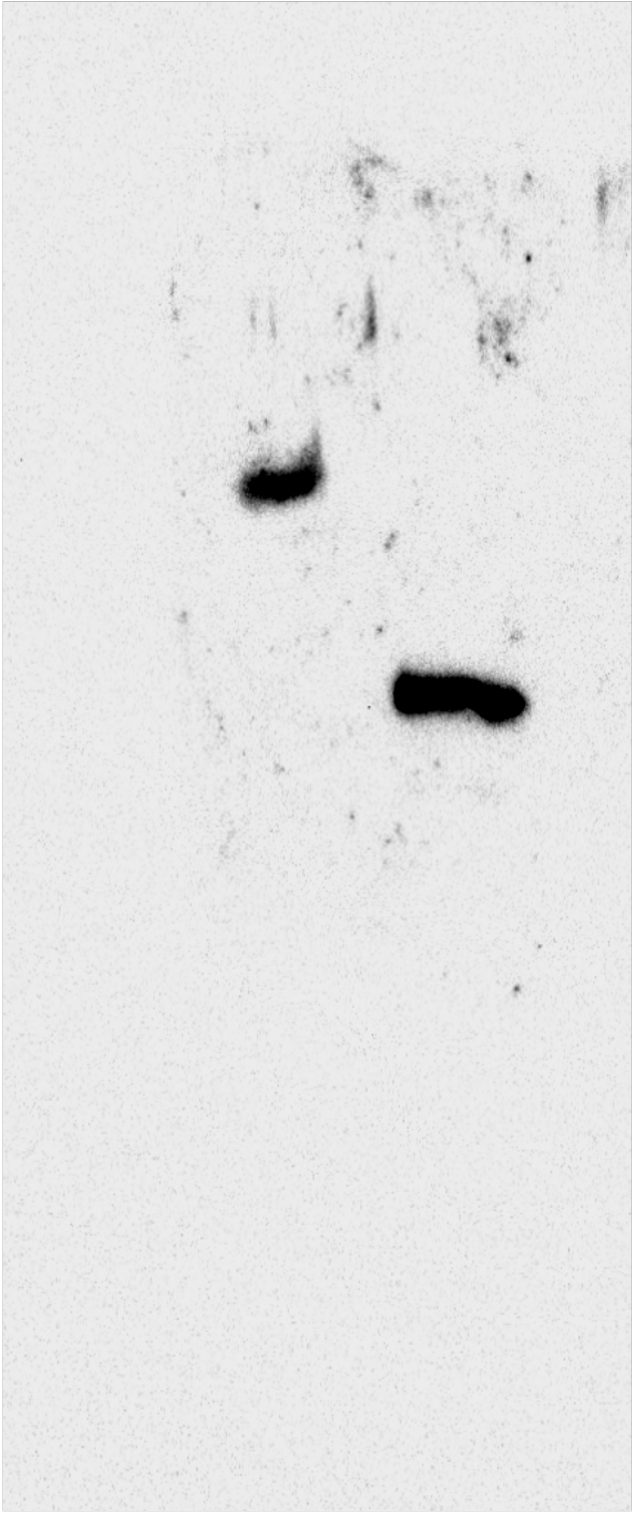

Supplement: Figure 1—figure supplement 1—source data 1. [file elife-96933-fig1-figsupp1-data1.zip › Figure 1-figure supplement 1-source data 1/Figure 1-figure supplement 1A-western.tif]

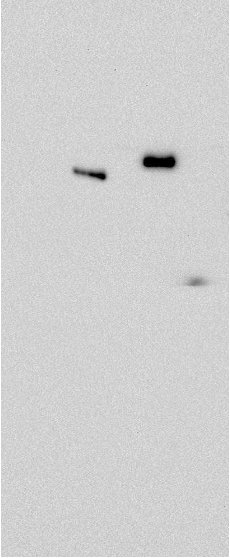

Supplement: Figure 1—figure supplement 1—source data 1. [file elife-96933-fig1-figsupp1-data1.zip › Figure 1-figure supplement 1-source data 1/Figure 1-figure supplement 1B-western.tif]

(A)

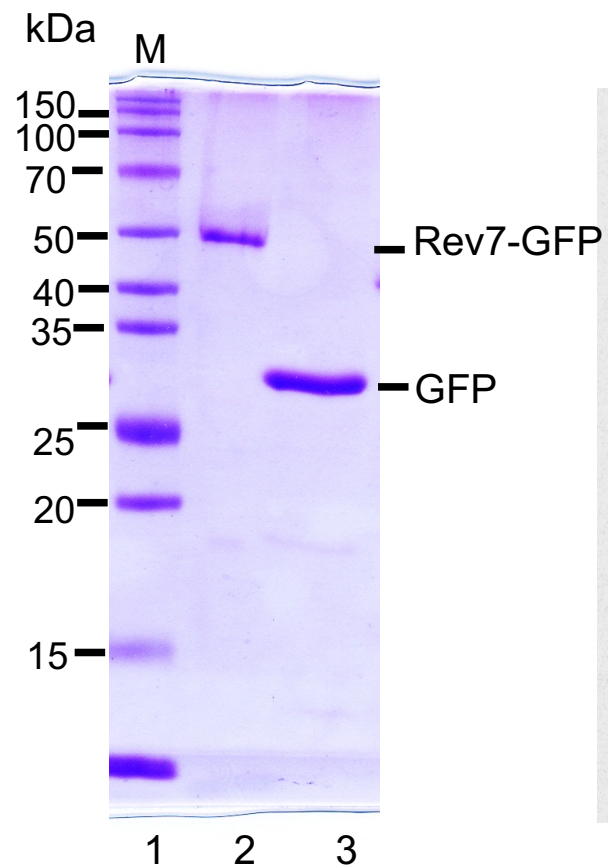

(B)

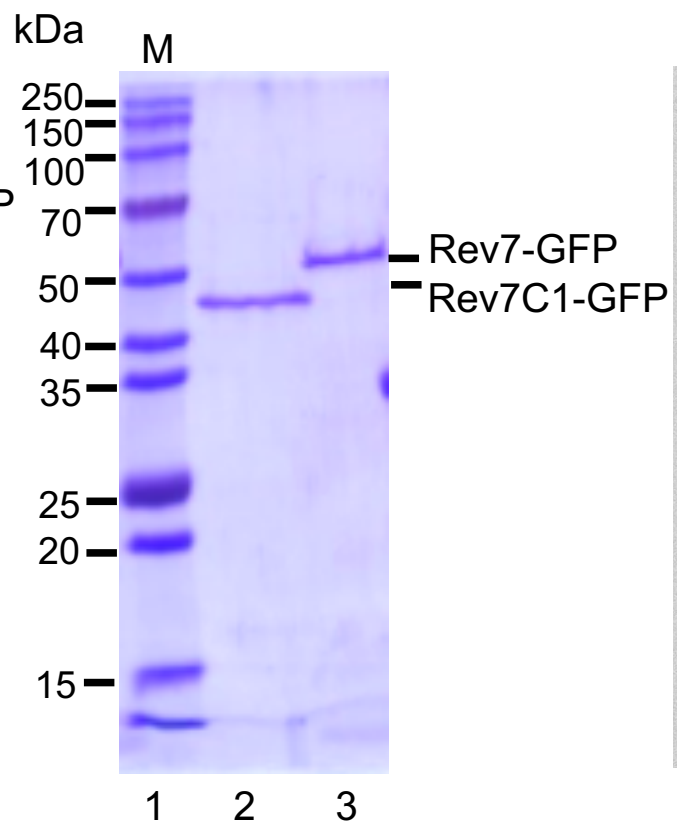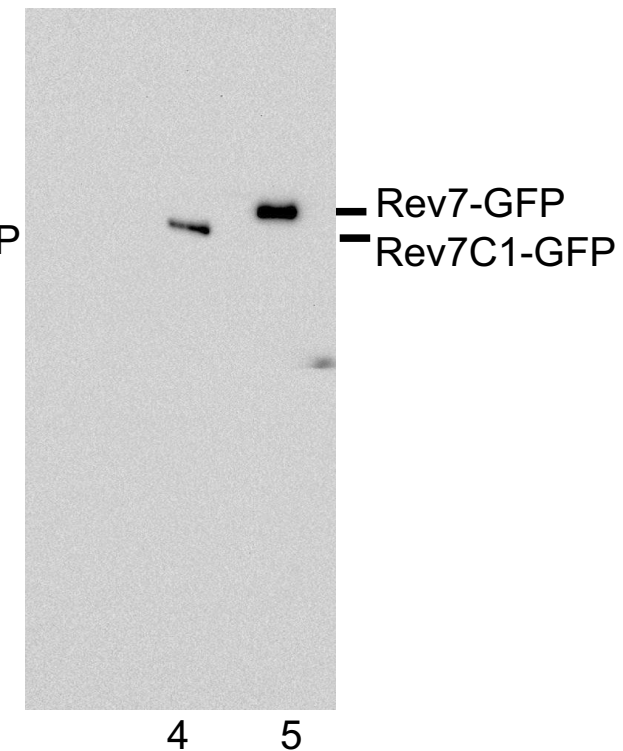

(C)

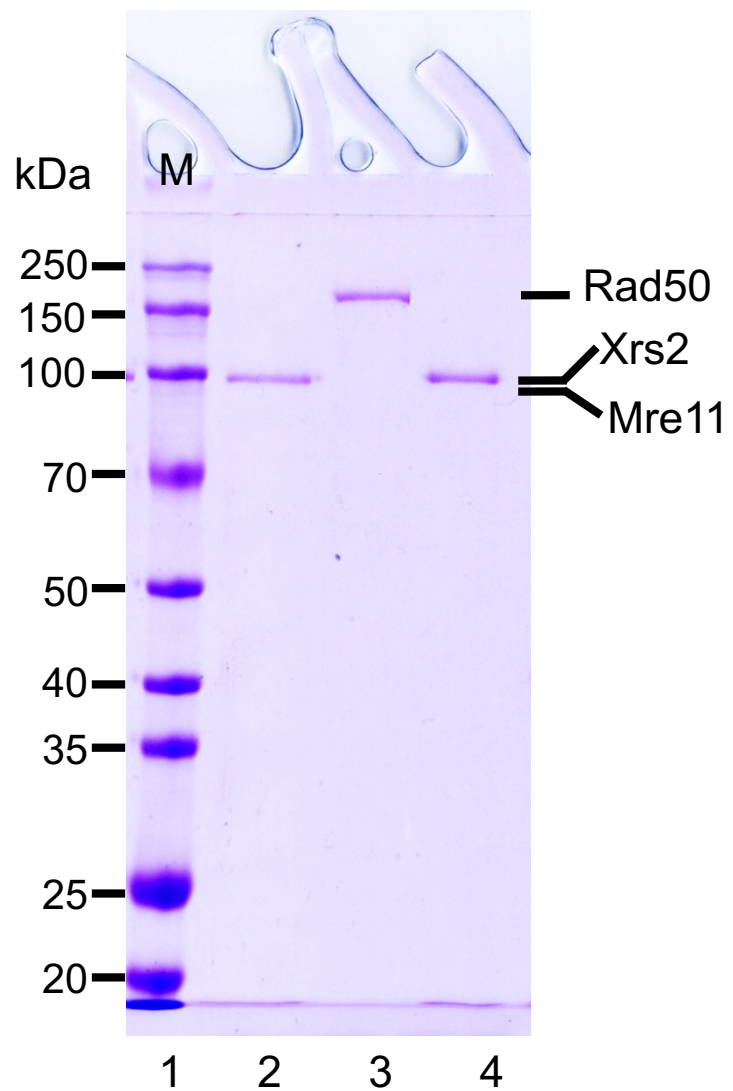

(D)

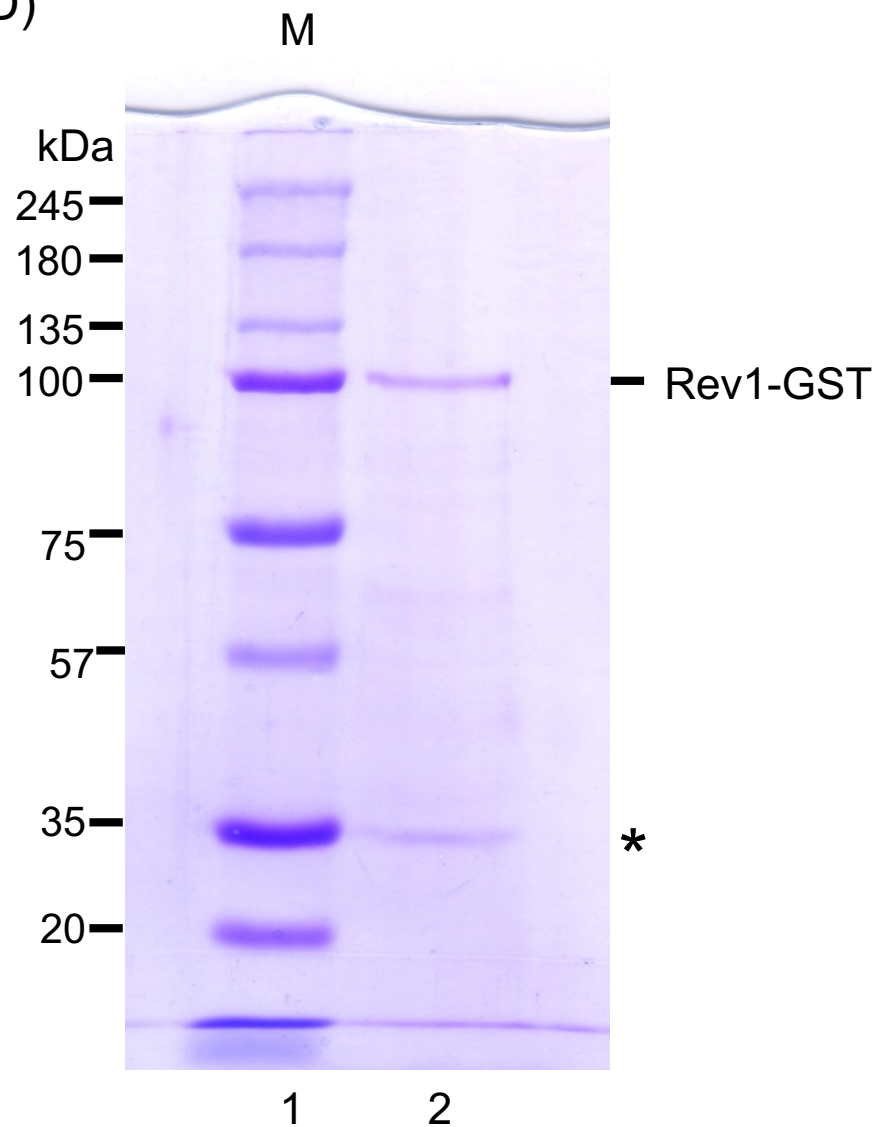

(E)

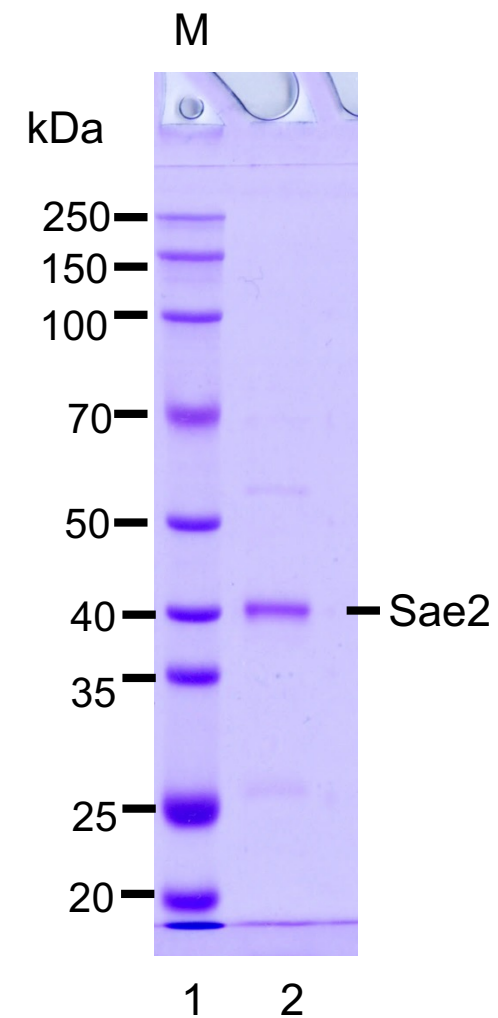

(F)

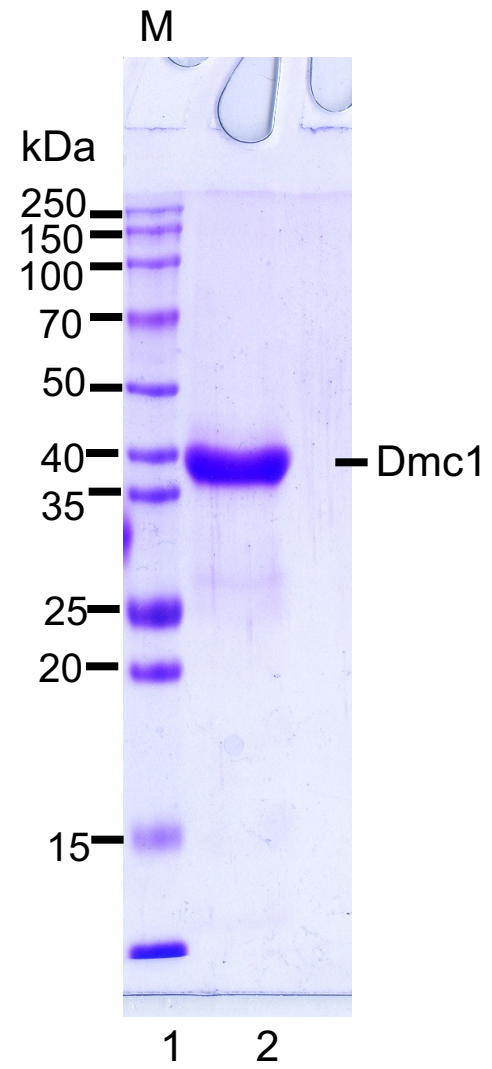

Supplement: Figure 1—figure supplement 1—source data 2. [file elife-96933-fig1-figsupp1-data2.zip › Figure 1-figure supplement 1-source data 2/Figure 1-figure supplement 1-source data 2.pdf]

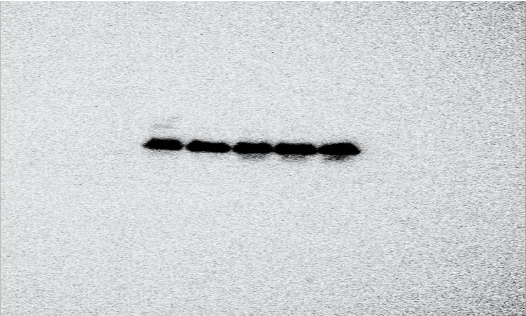

Supplement: Figure 2—figure supplement 2—source data 1. [file elife-96933-fig2-figsupp2-data1.zip › Figure 2-figure supplement 2-source data 1/Rev7-N1.tif]

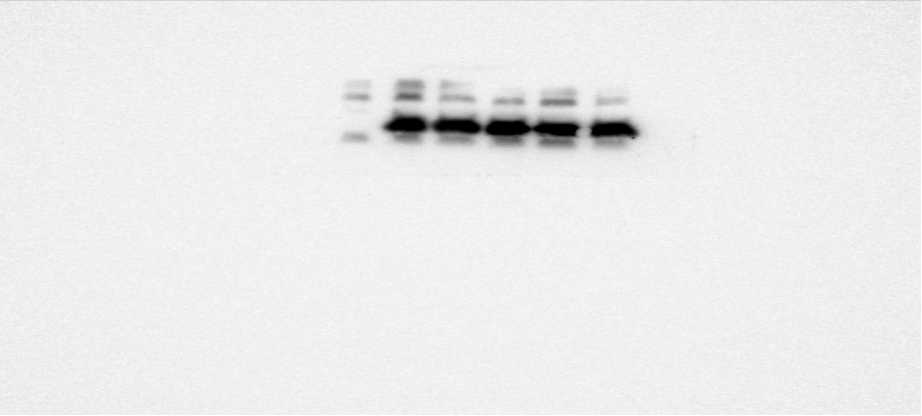

Supplement: Figure 2—figure supplement 2—source data 1. [file elife-96933-fig2-figsupp2-data1.zip › Figure 2-figure supplement 2-source data 1/Rev7-N2.tif]

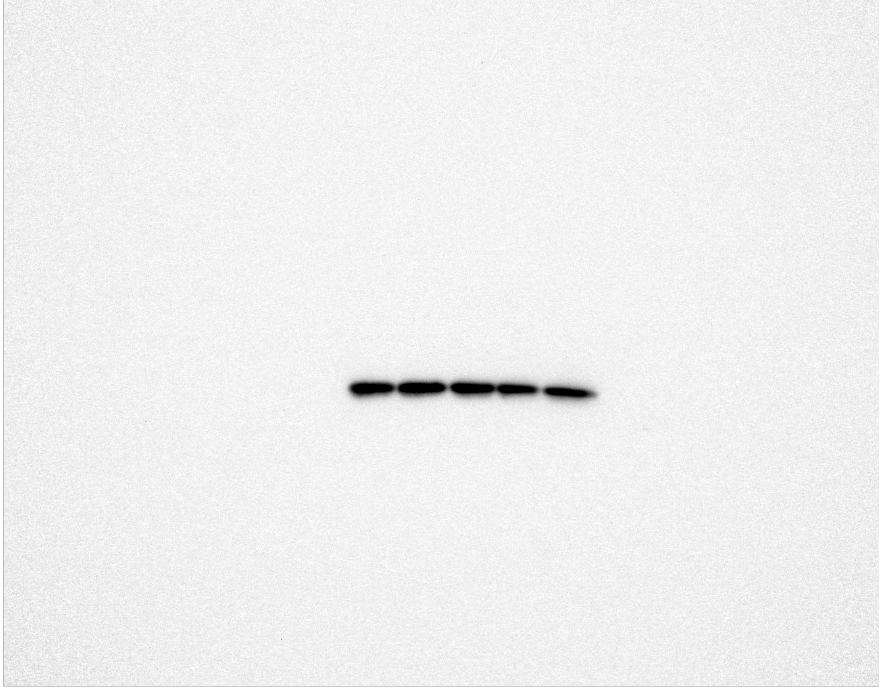

Supplement: Figure 2—figure supplement 2—source data 1. [file elife-96933-fig2-figsupp2-data1.zip › Figure 2-figure supplement 2-source data 1/Rev7-N3.tif]

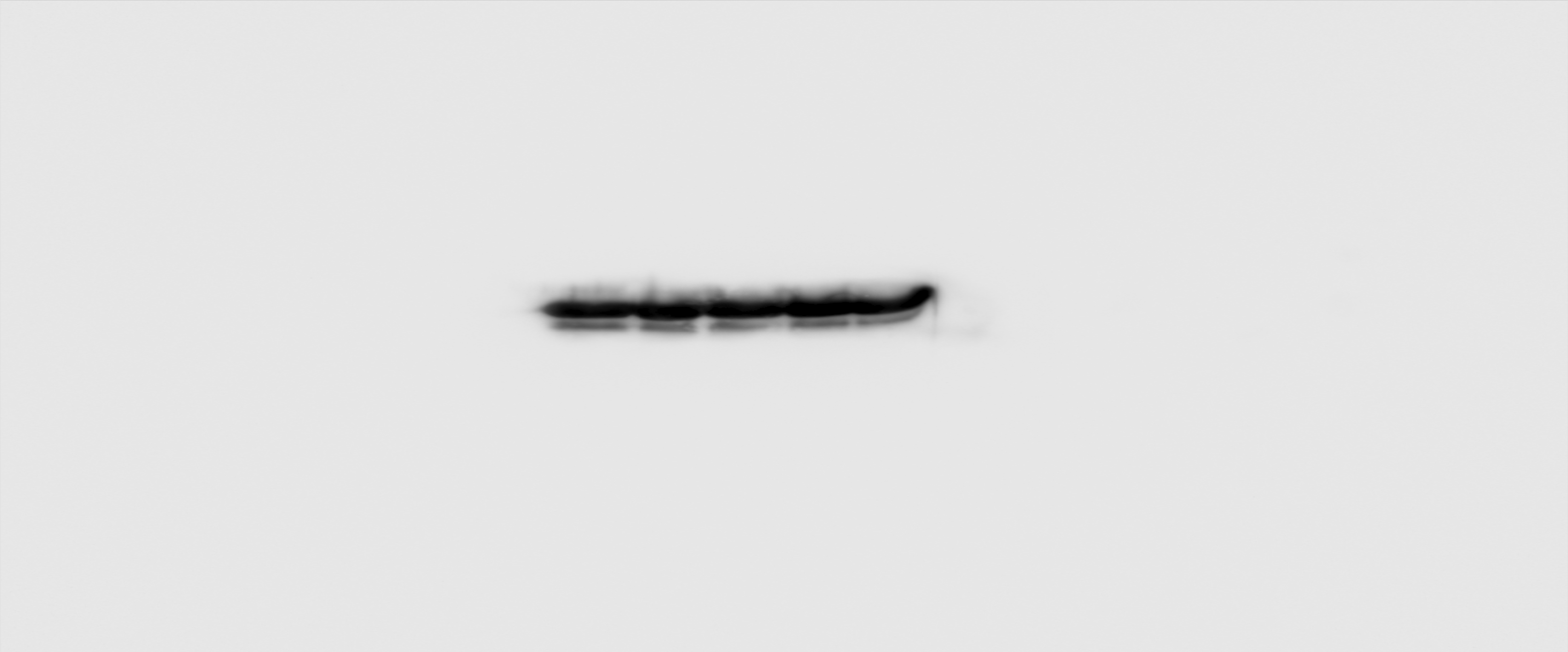

Supplement: Figure 2—figure supplement 2—source data 1. [file elife-96933-fig2-figsupp2-data1.zip › Figure 2-figure supplement 2-source data 1/Rev7-C1.tif]

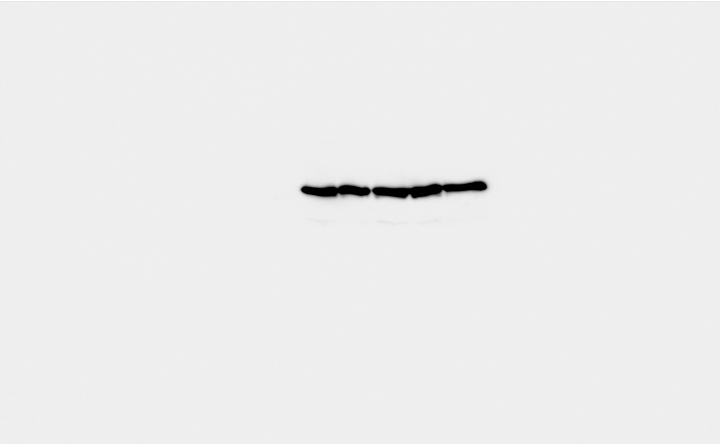

Supplement: Figure 2—figure supplement 2—source data 1. [file elife-96933-fig2-figsupp2-data1.zip › Figure 2-figure supplement 2-source data 1/Rev7-C3.tif]

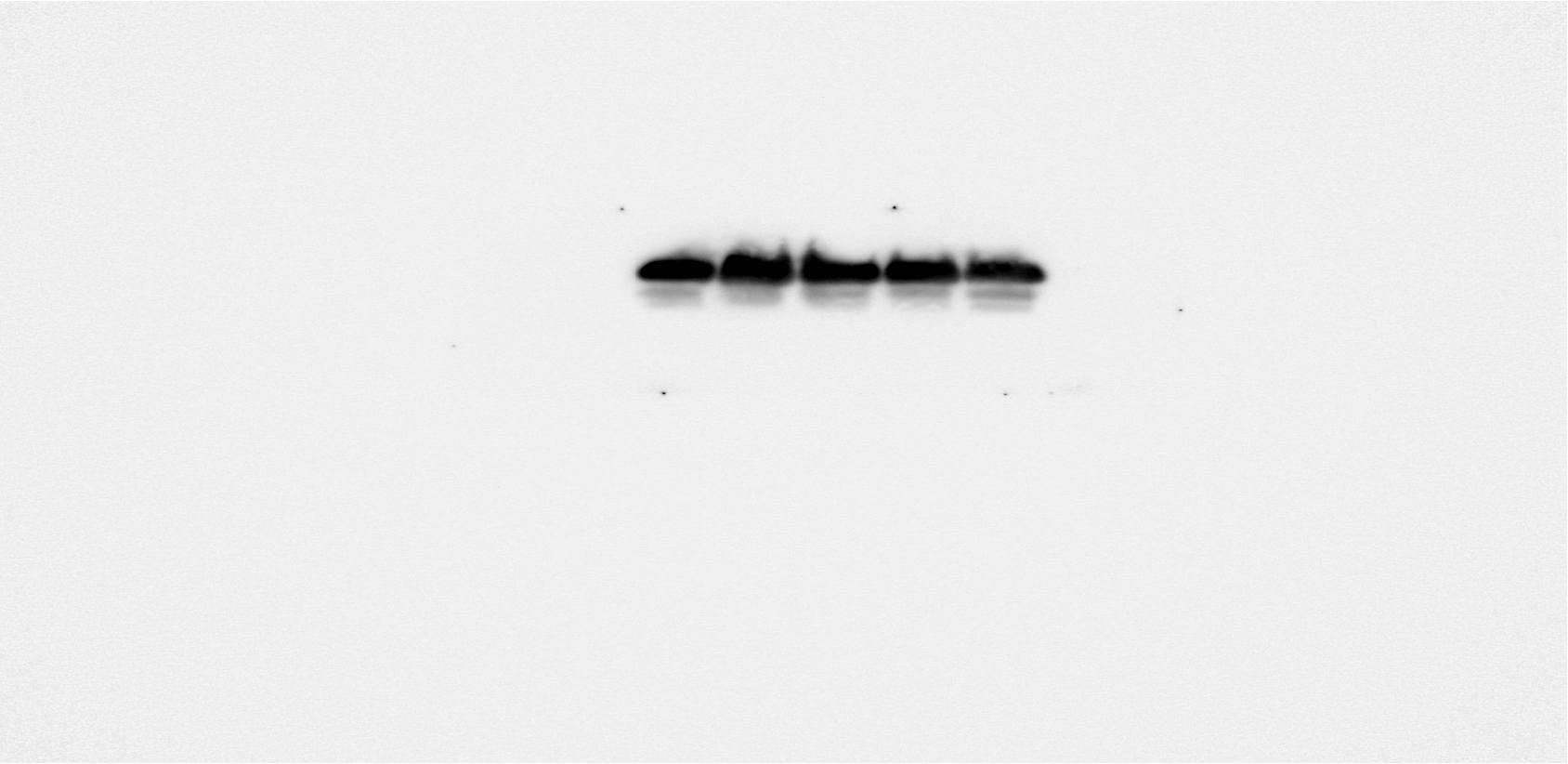

Supplement: Figure 2—figure supplement 2—source data 1. [file elife-96933-fig2-figsupp2-data1.zip › Figure 2-figure supplement 2-source data 1/Rev7-C2.tif]

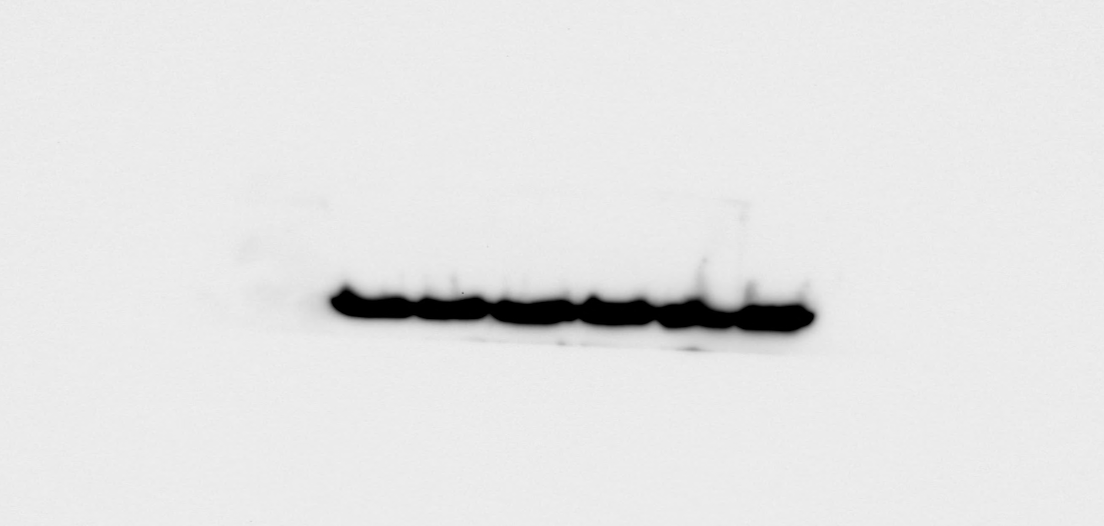

Supplement: Figure 2—figure supplement 2—source data 1. [file elife-96933-fig2-figsupp2-data1.zip › Figure 2-figure supplement 2-source data 1/Pgk1.tif]

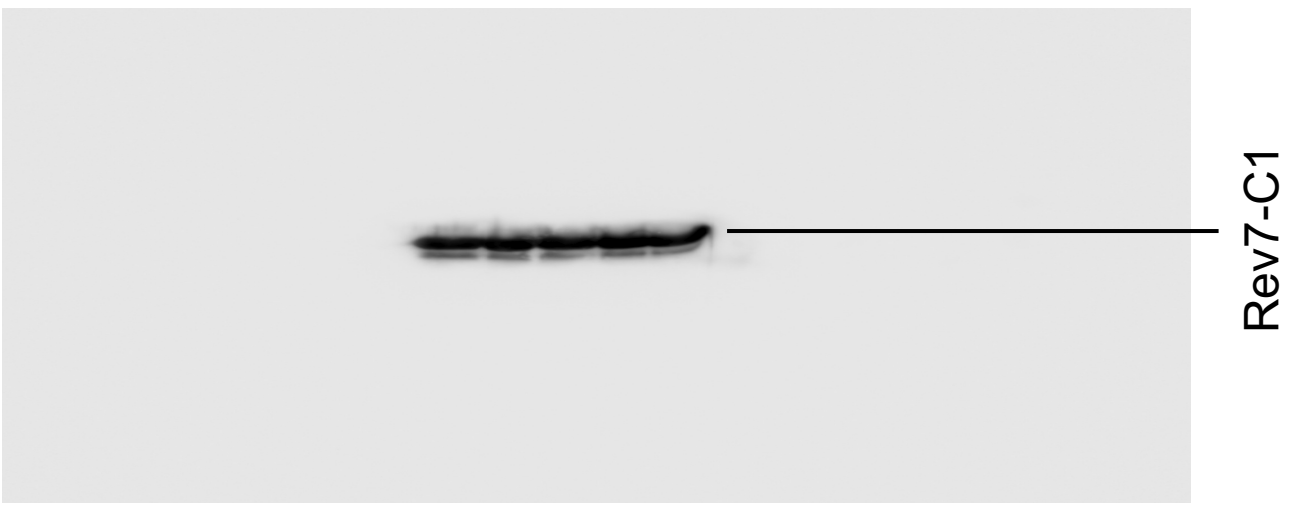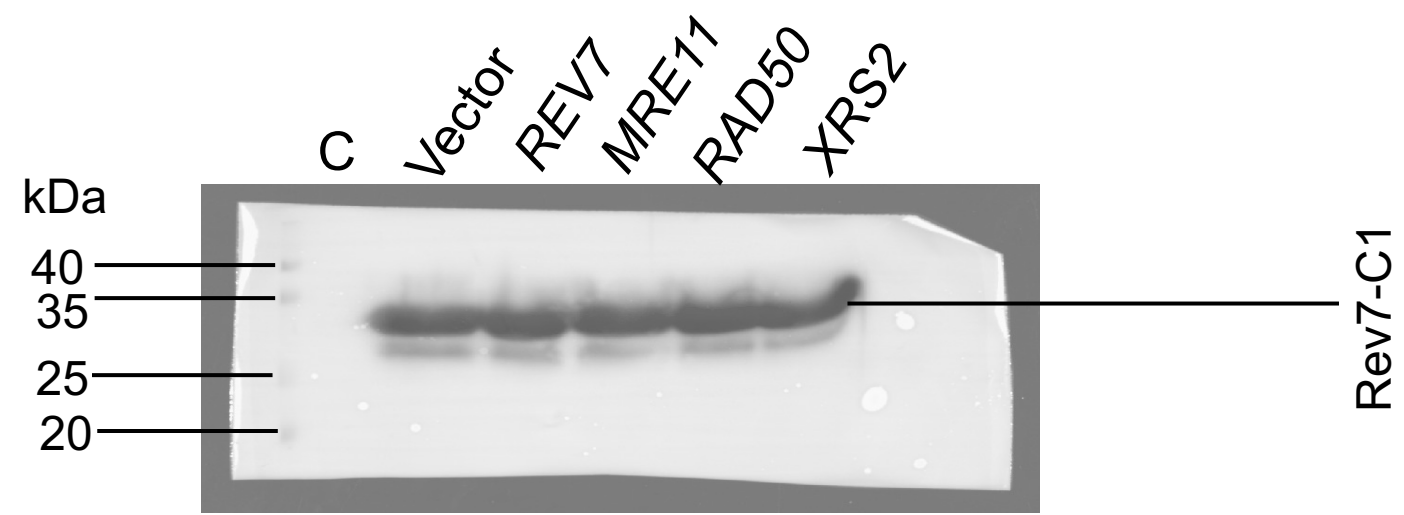

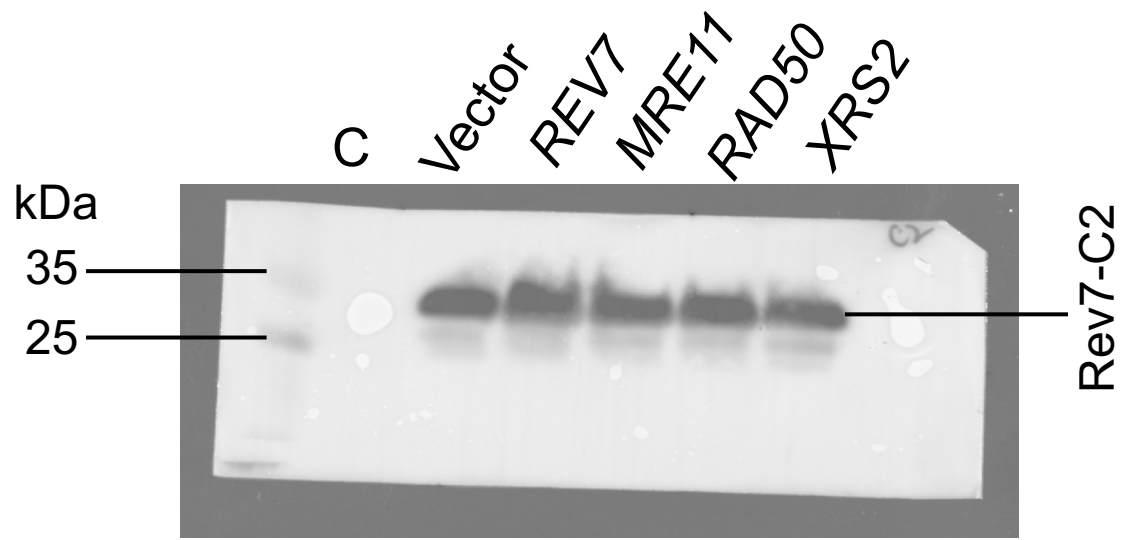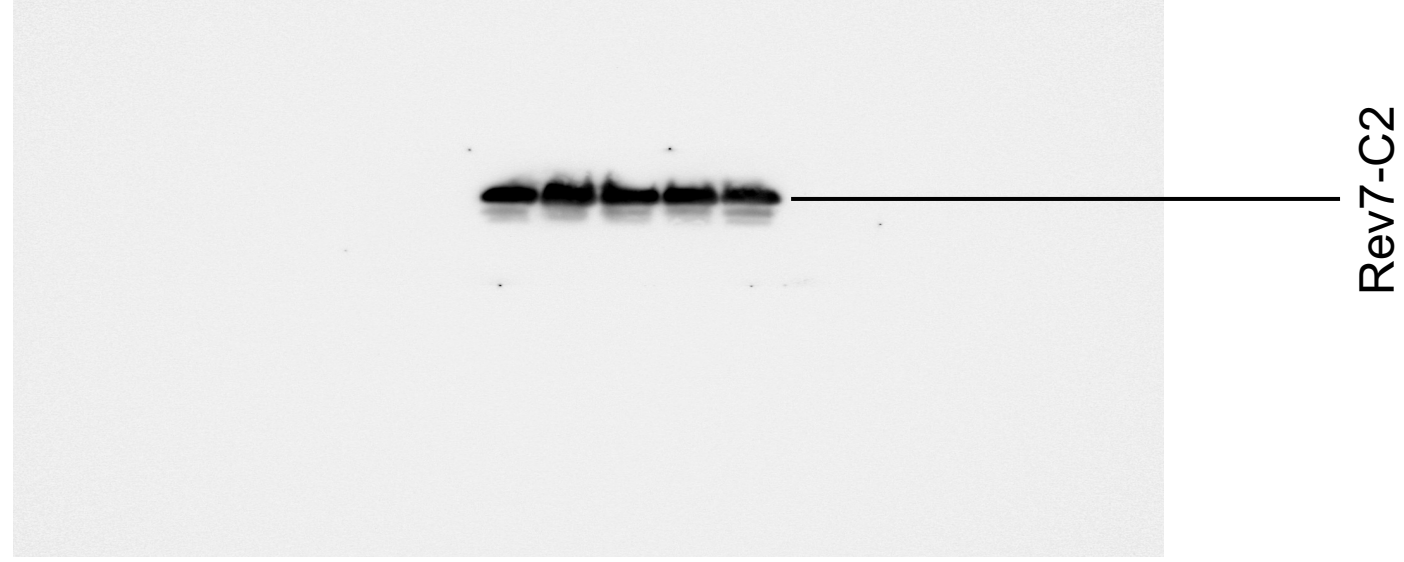

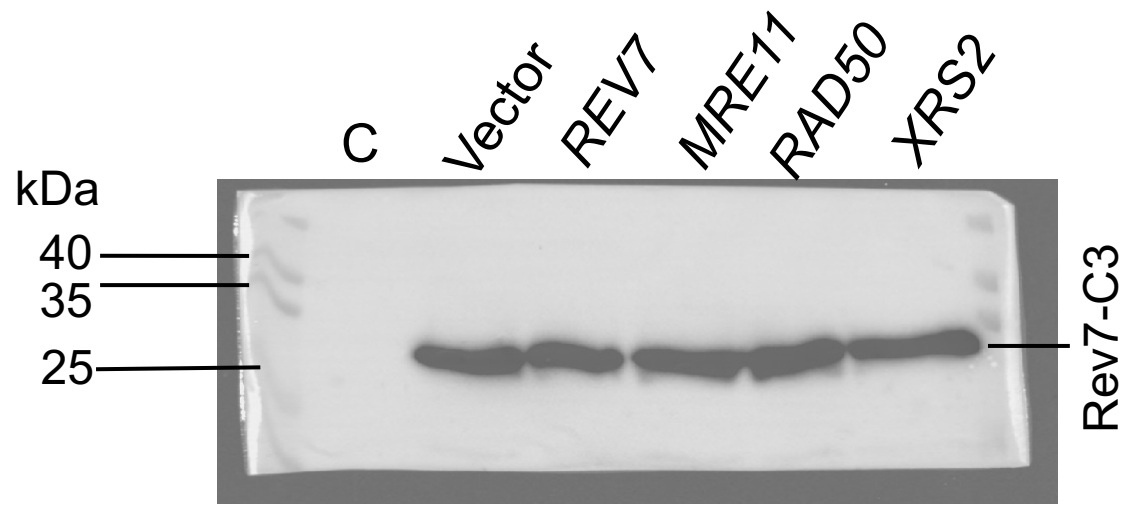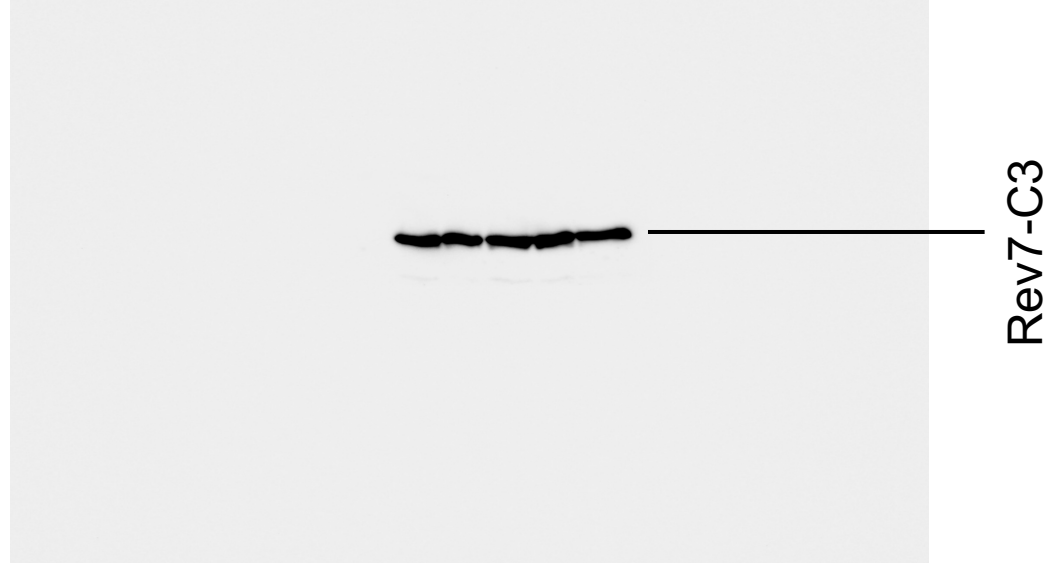

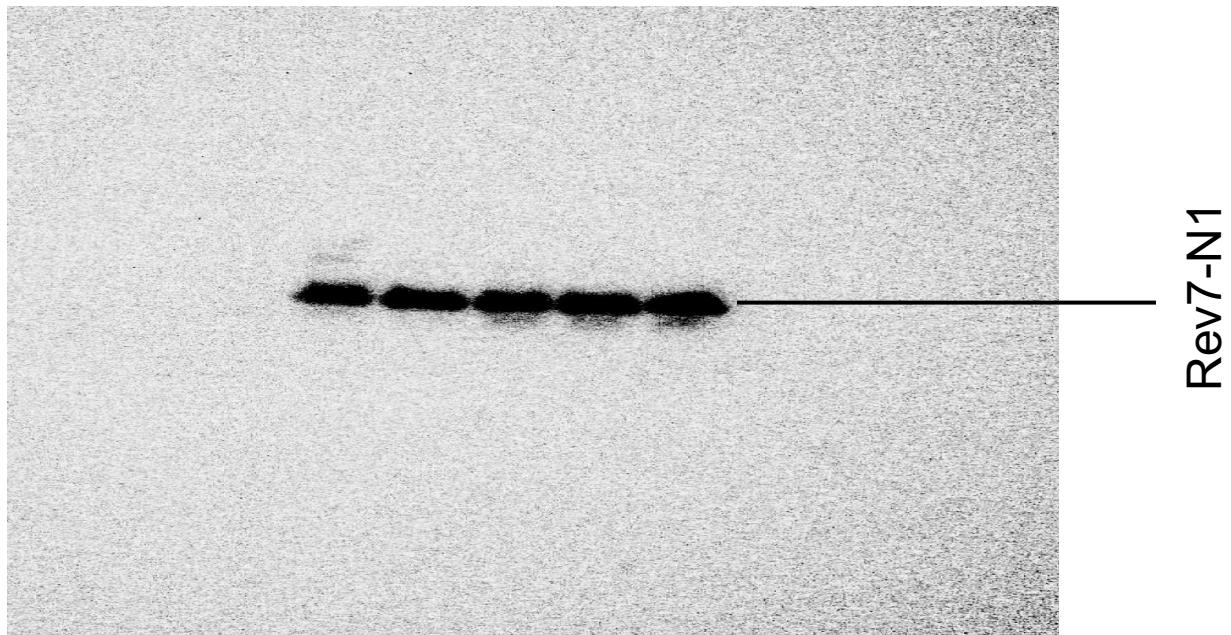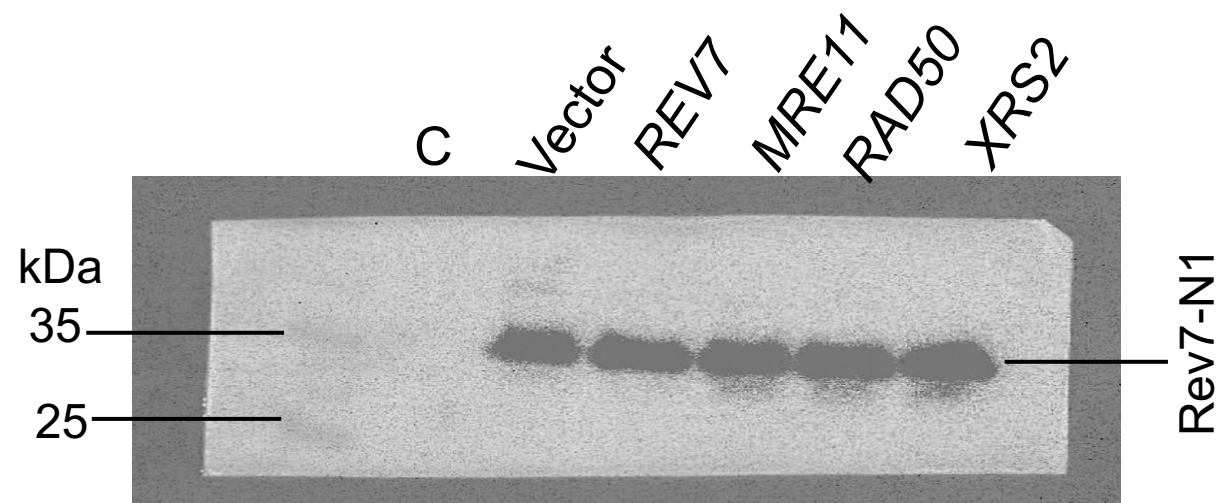

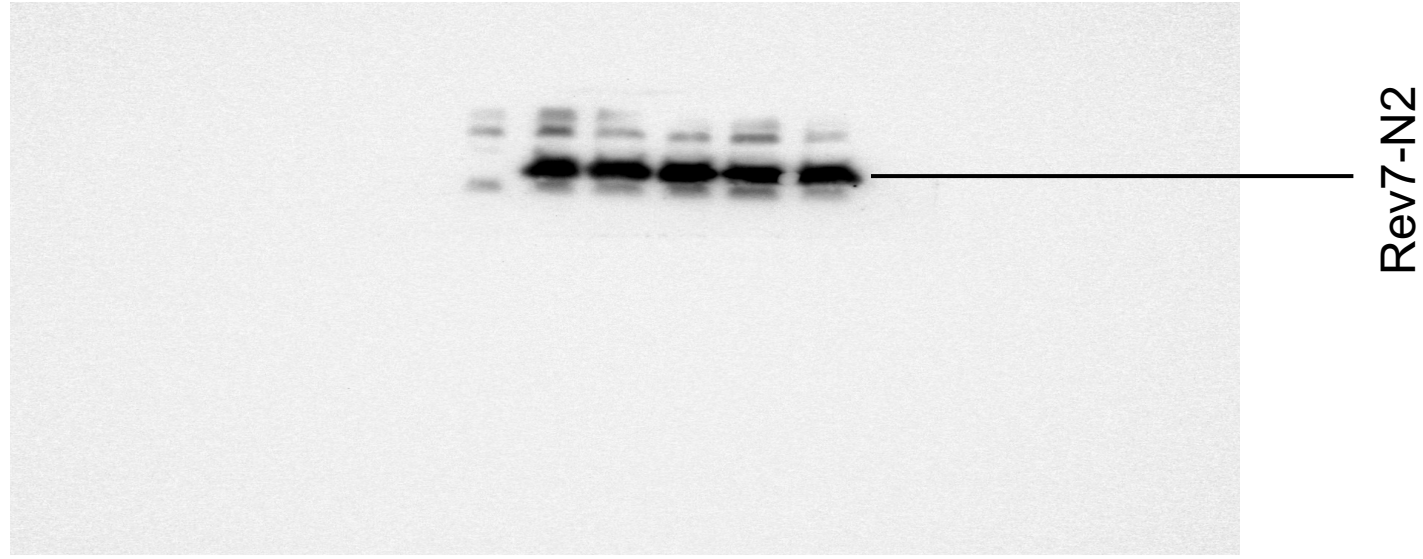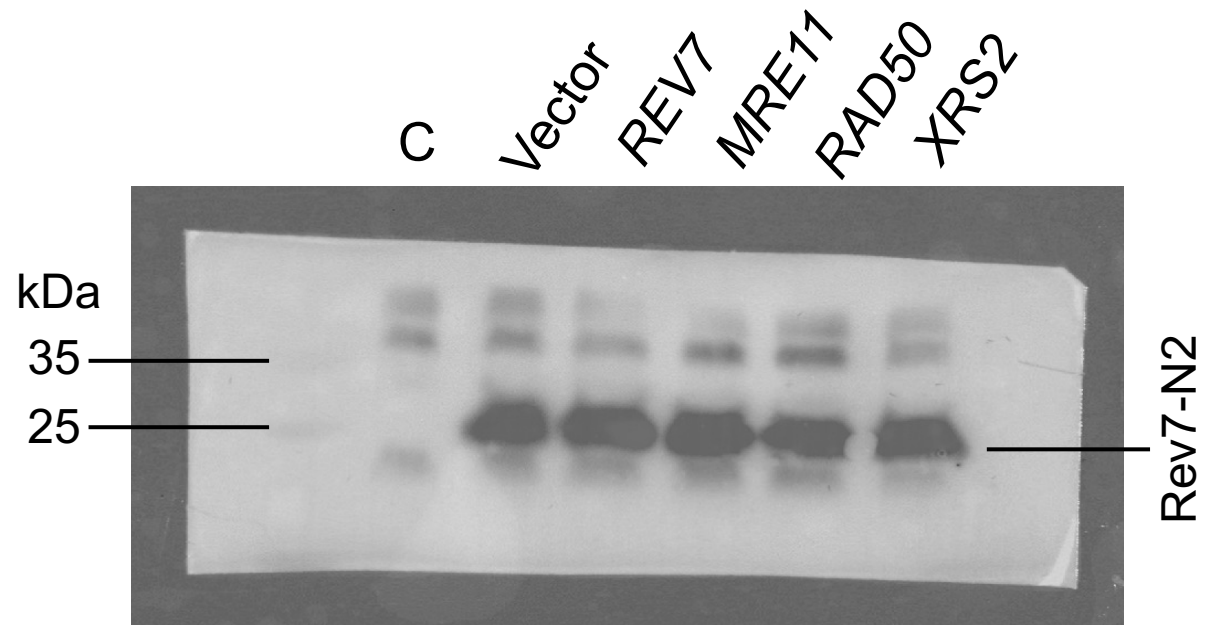

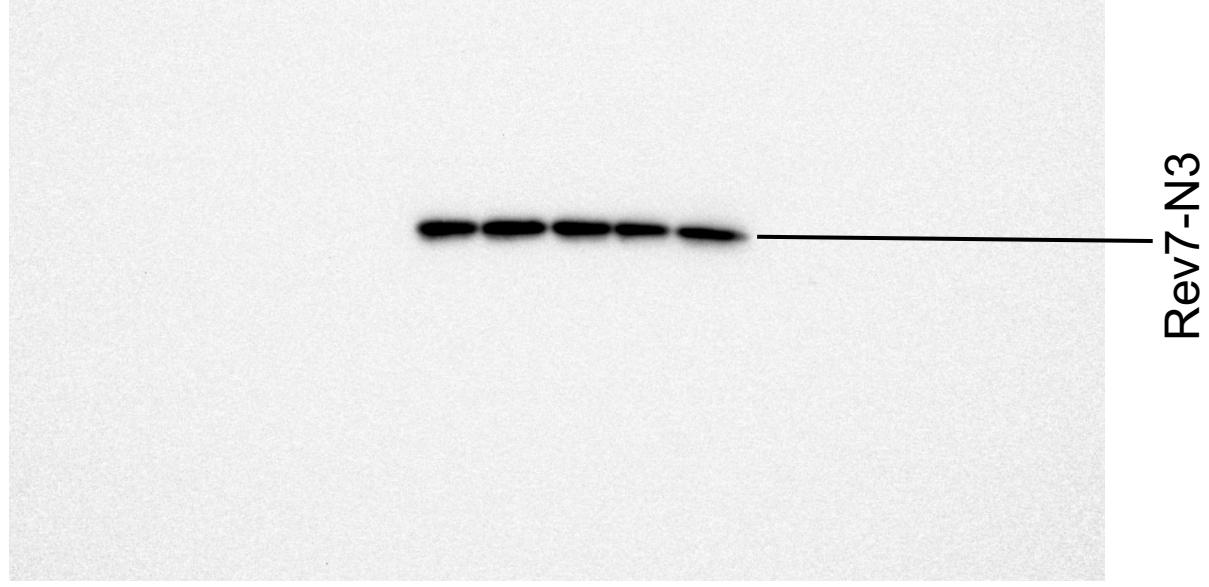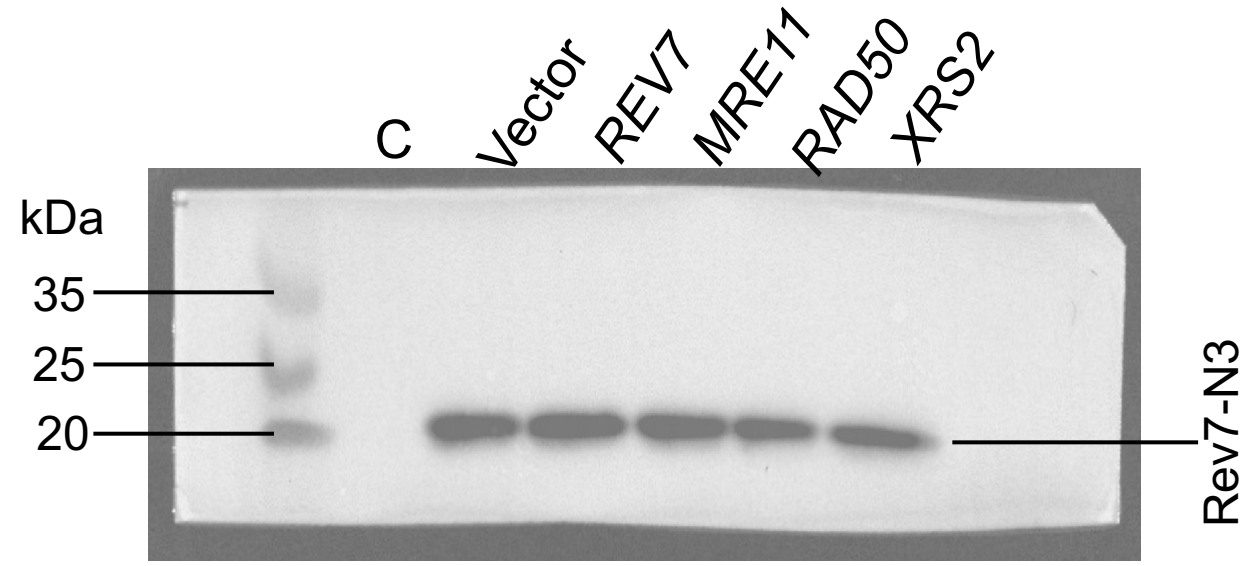

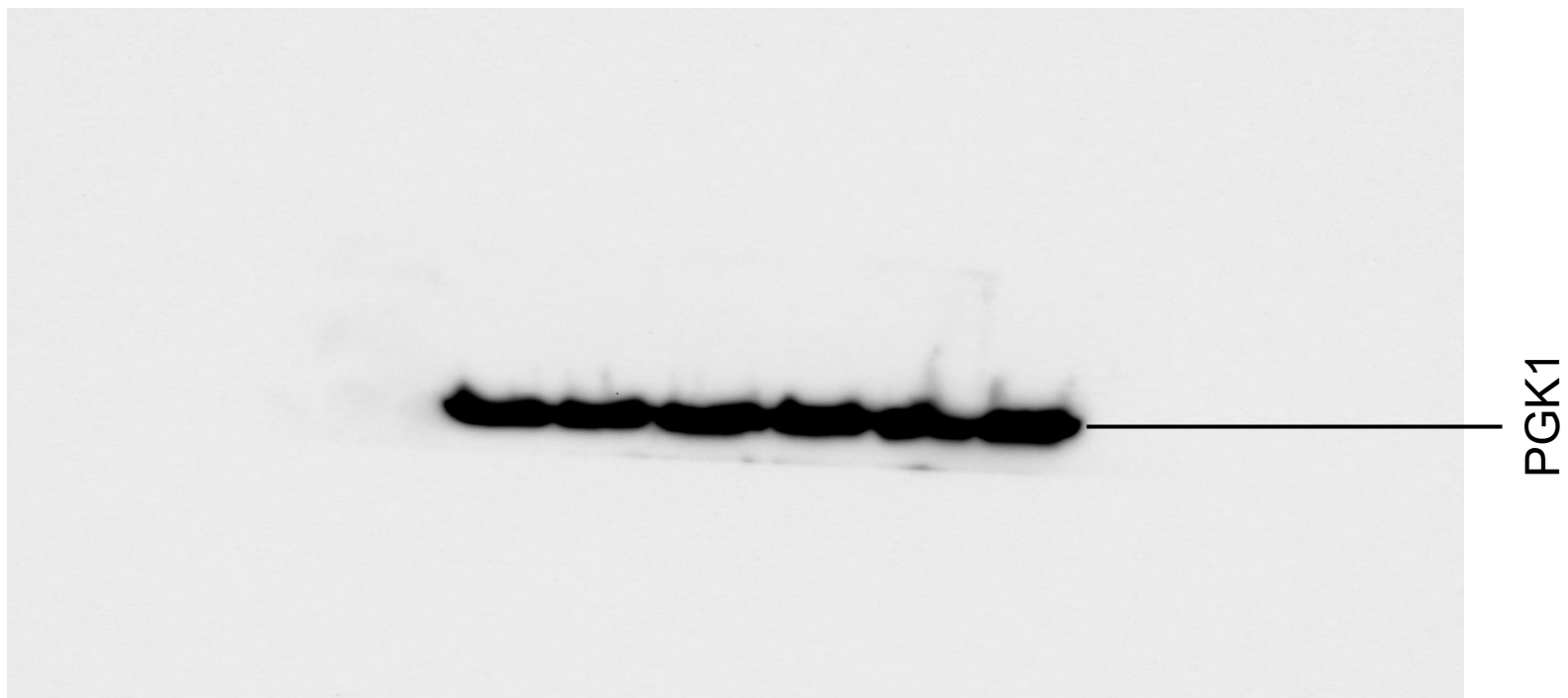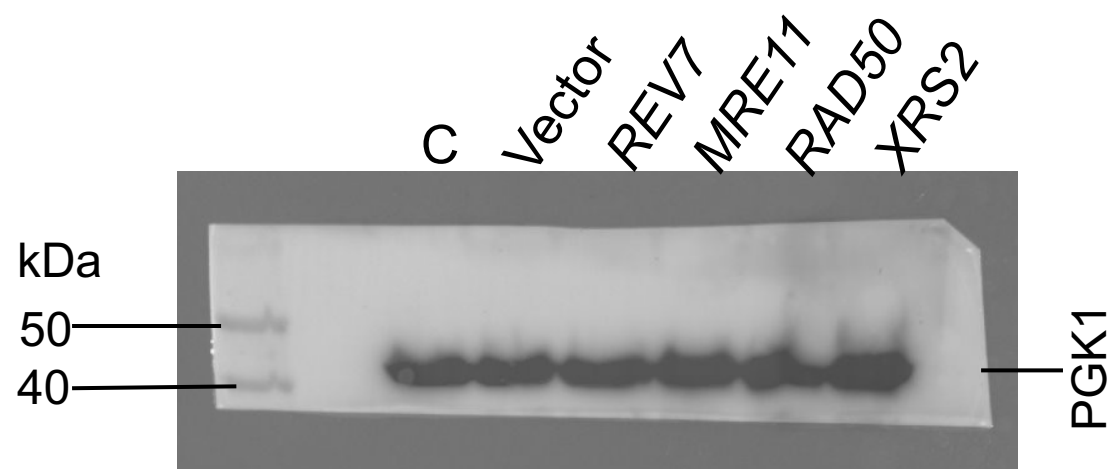

Supplement: Figure 2—figure supplement 2—source data 2. [file elife-96933-fig2-figsupp2-data2.zip › Figure 2-figure supplement 2-source data 2/Figure 2-figure supplement 2-source data.pdf]

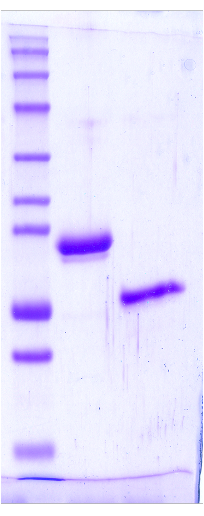

Supplement: Figure 4—source data 1. [file elife-96933-fig4-data1.zip › Figure 4-source data 1/Figure 4B-source data.tif]

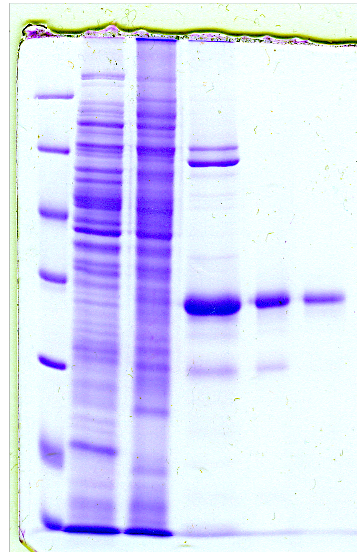

Supplement: Figure 4—source data 1. [file elife-96933-fig4-data1.zip › Figure 4-source data 1/Figure 4A-source data.tif]

(A)

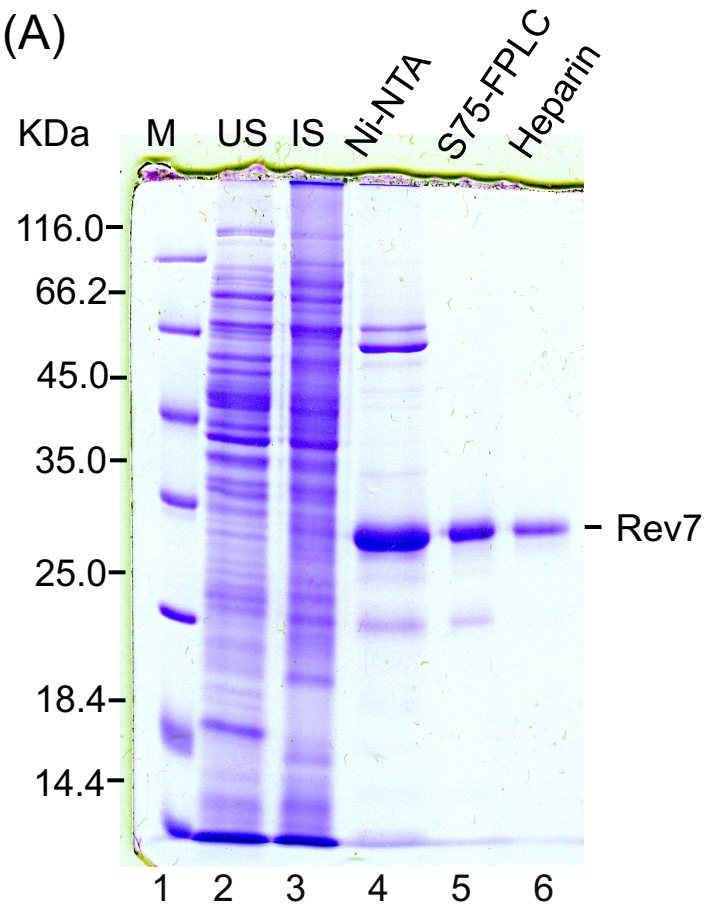

(B)

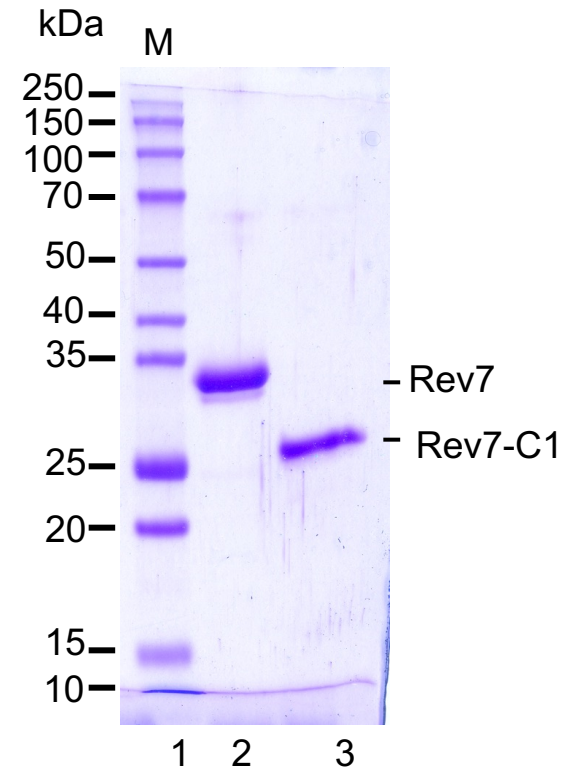

Supplement: Figure 4—source data 2. [file elife-96933-fig4-data2.zip › Figure 4-source data 2/Figure 4-source data.pdf]

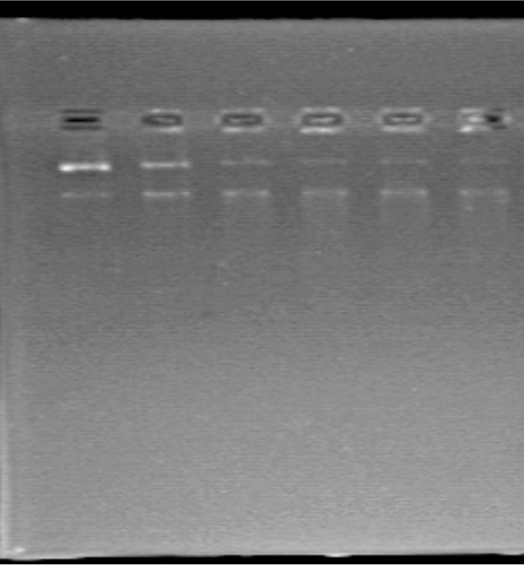

Supplement: Figure 5—source data 1. [file elife-96933-fig5-data1.zip › Figure 5-source data 1/Source data-figure 5D.tif]

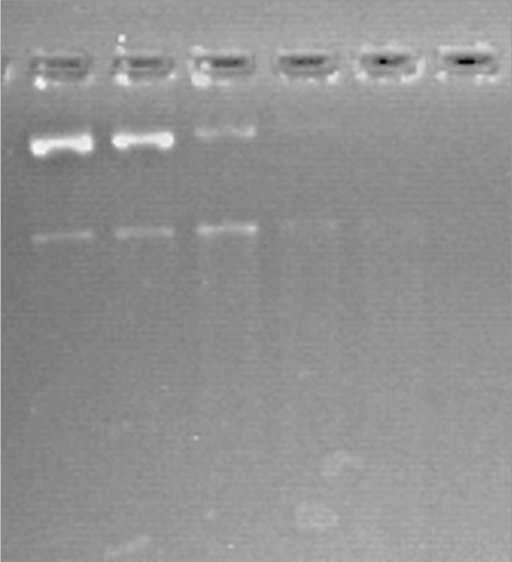

Supplement: Figure 5—source data 1. [file elife-96933-fig5-data1.zip › Figure 5-source data 1/Source data-figure 5C.tif]

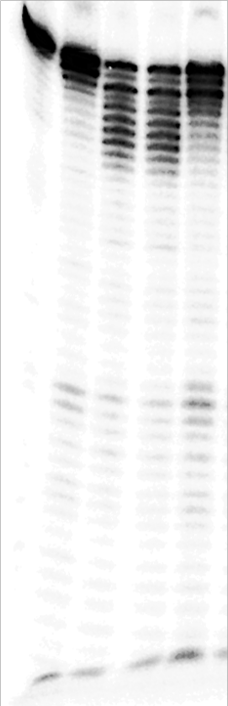

Supplement: Figure 5—source data 1. [file elife-96933-fig5-data1.zip › Figure 5-source data 1/Source data-Figure 5B.tif]

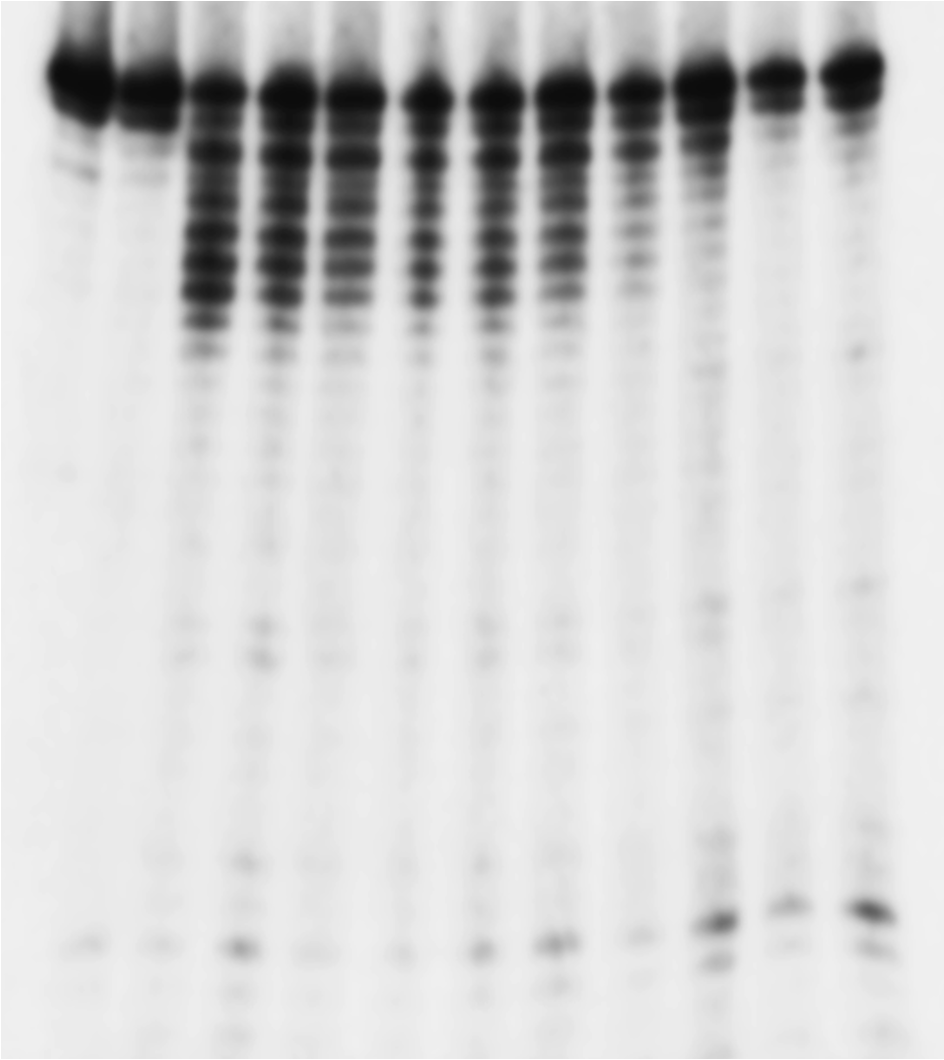

Supplement: Figure 5—source data 1. [file elife-96933-fig5-data1.zip › Figure 5-source data 1/Source data-Figure 5A.tif]

Figure 5:

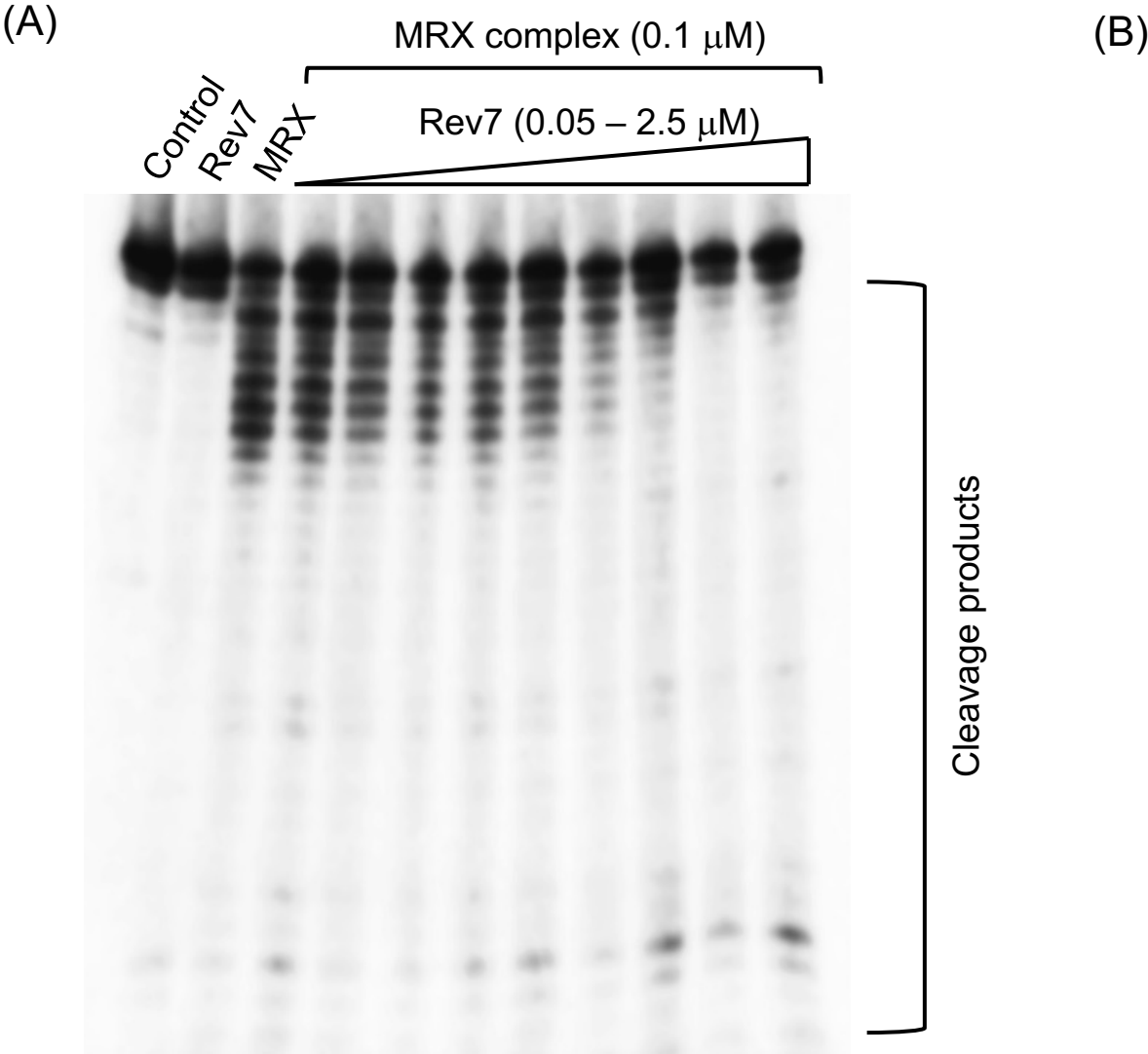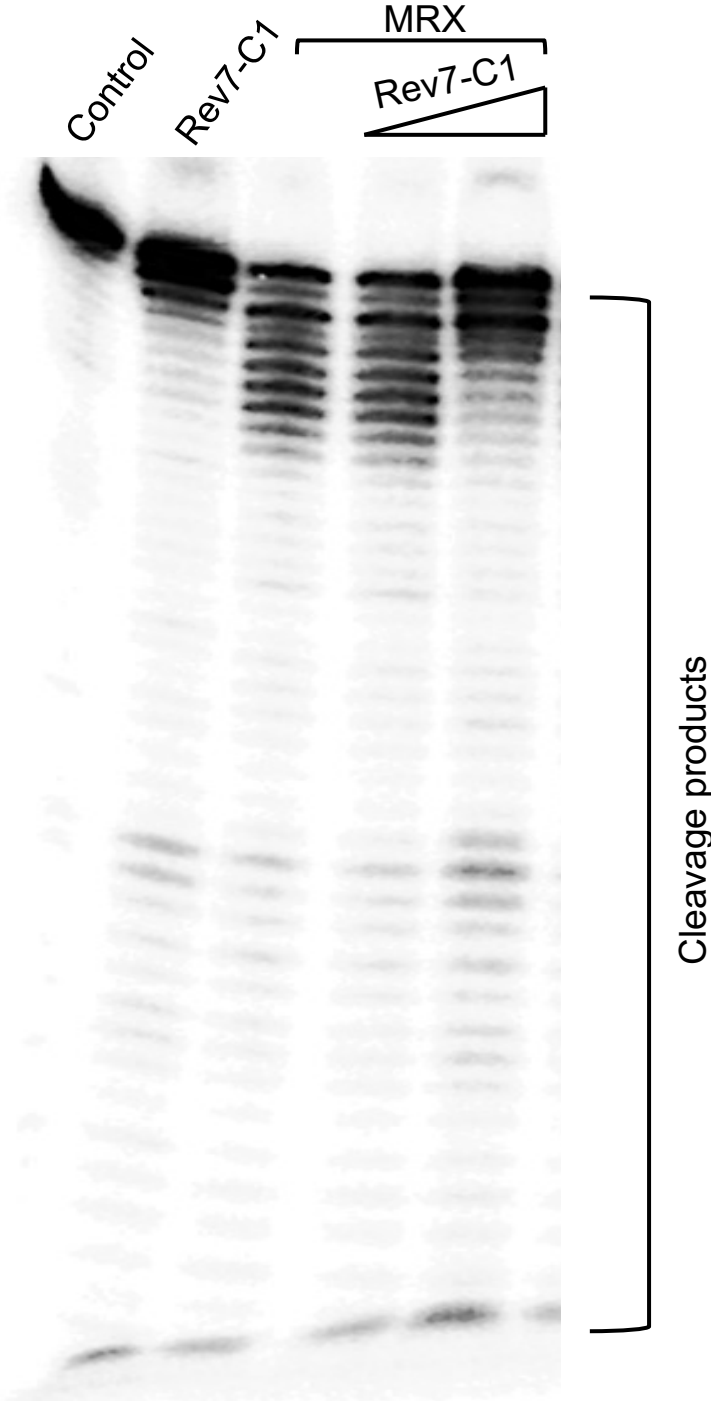

(C)

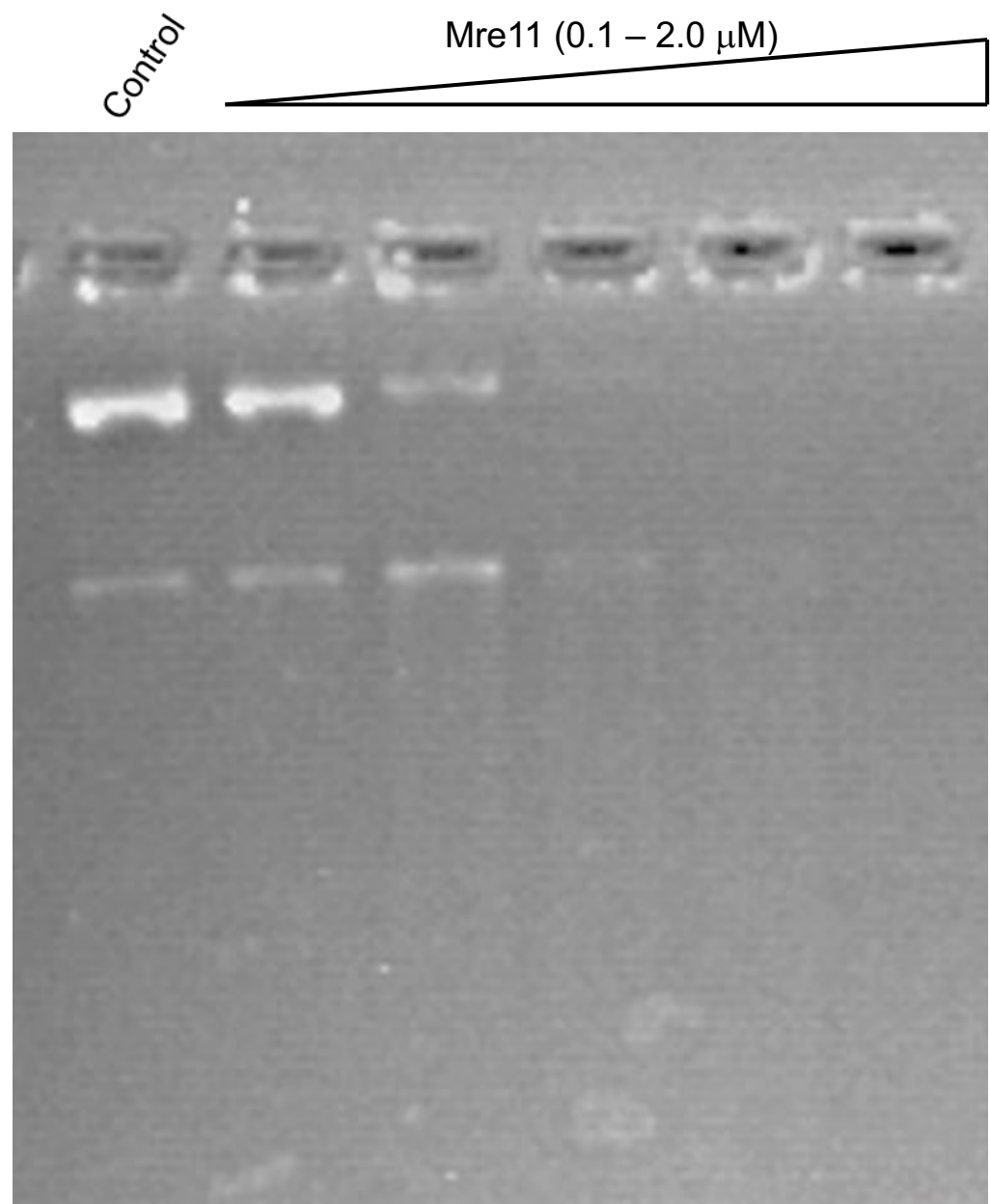

Circular  
ssDNA

Linear  
ssDNA

(D)

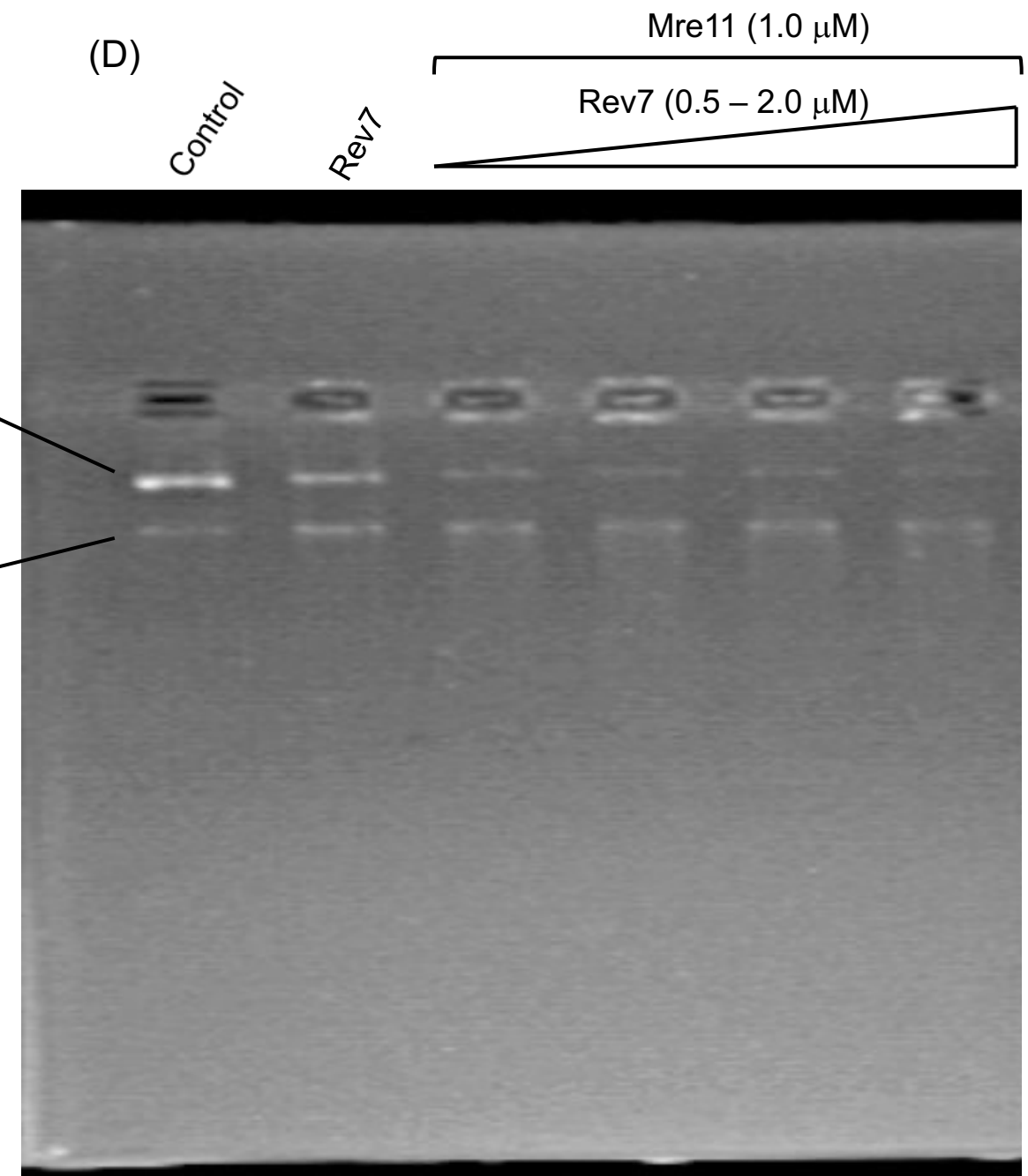

Supplement: Figure 5—source data 2. [file elife-96933-fig5-data2.zip › Figure 5-source data 2/Figure 5-source data 2.pdf]

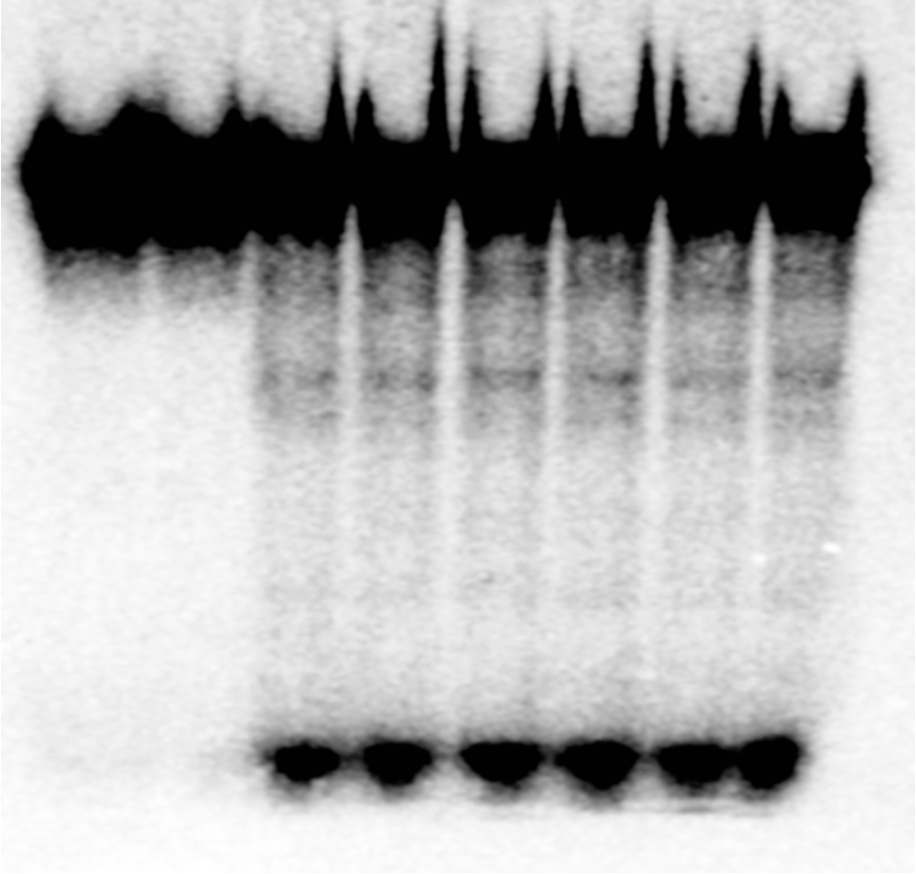

Supplement: Figure 5—figure supplement 1—source data 1. [file elife-96933-fig5-figsupp1-data1.zip › Figure 5-figure supplement 1-source data 1/Figure 5-figure supplement 1B.tif]

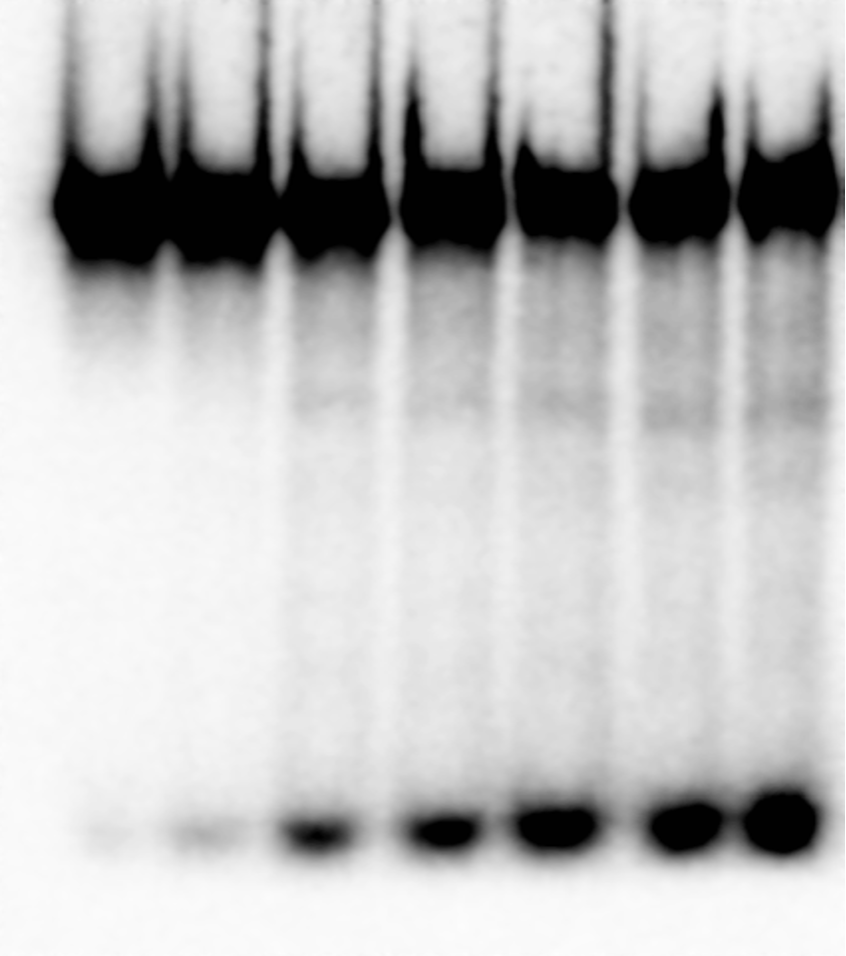

Supplement: Figure 5—figure supplement 1—source data 1. [file elife-96933-fig5-figsupp1-data1.zip › Figure 5-figure supplement 1-source data 1/Figure 5-figure supplement 1A.TIF]

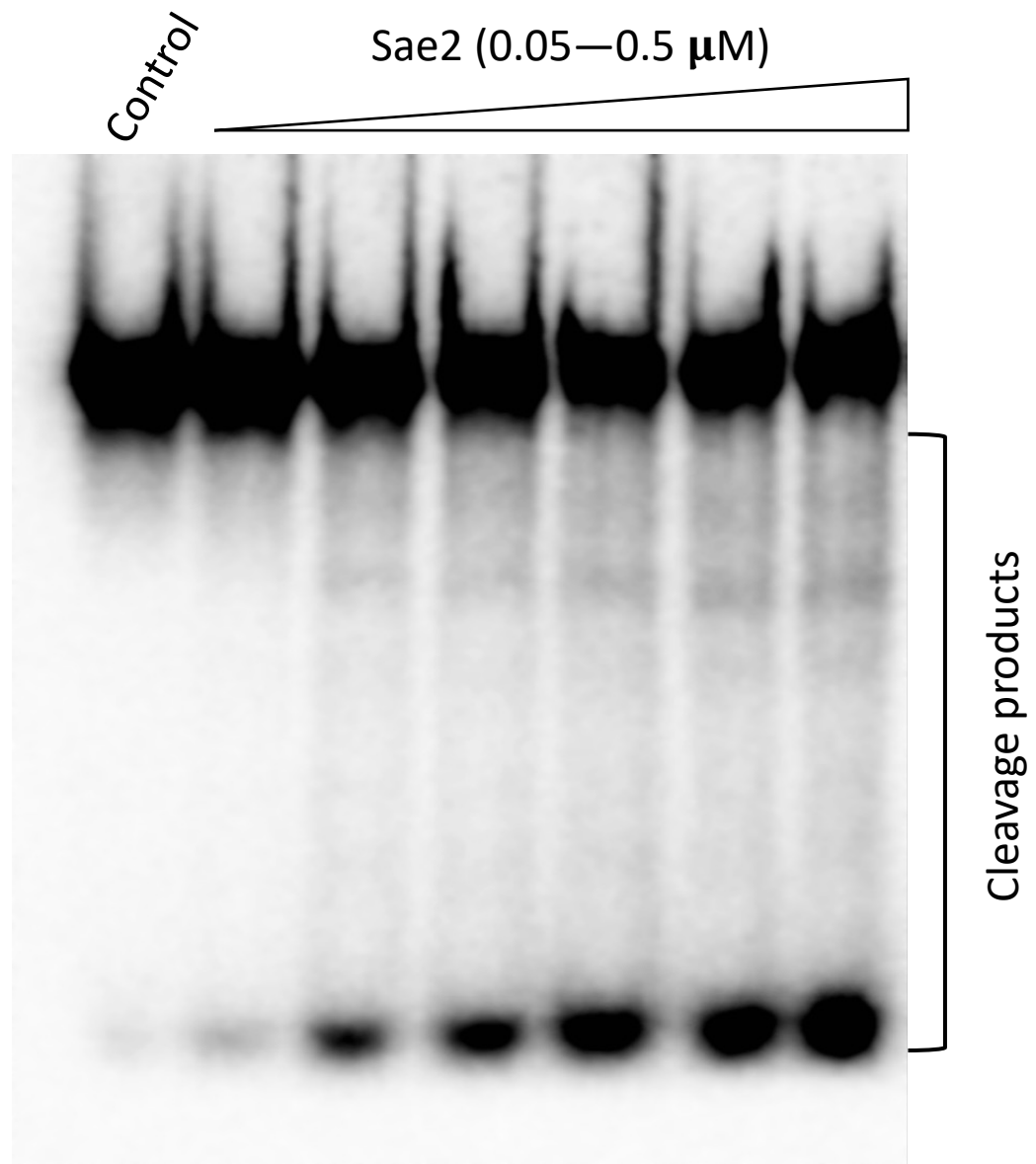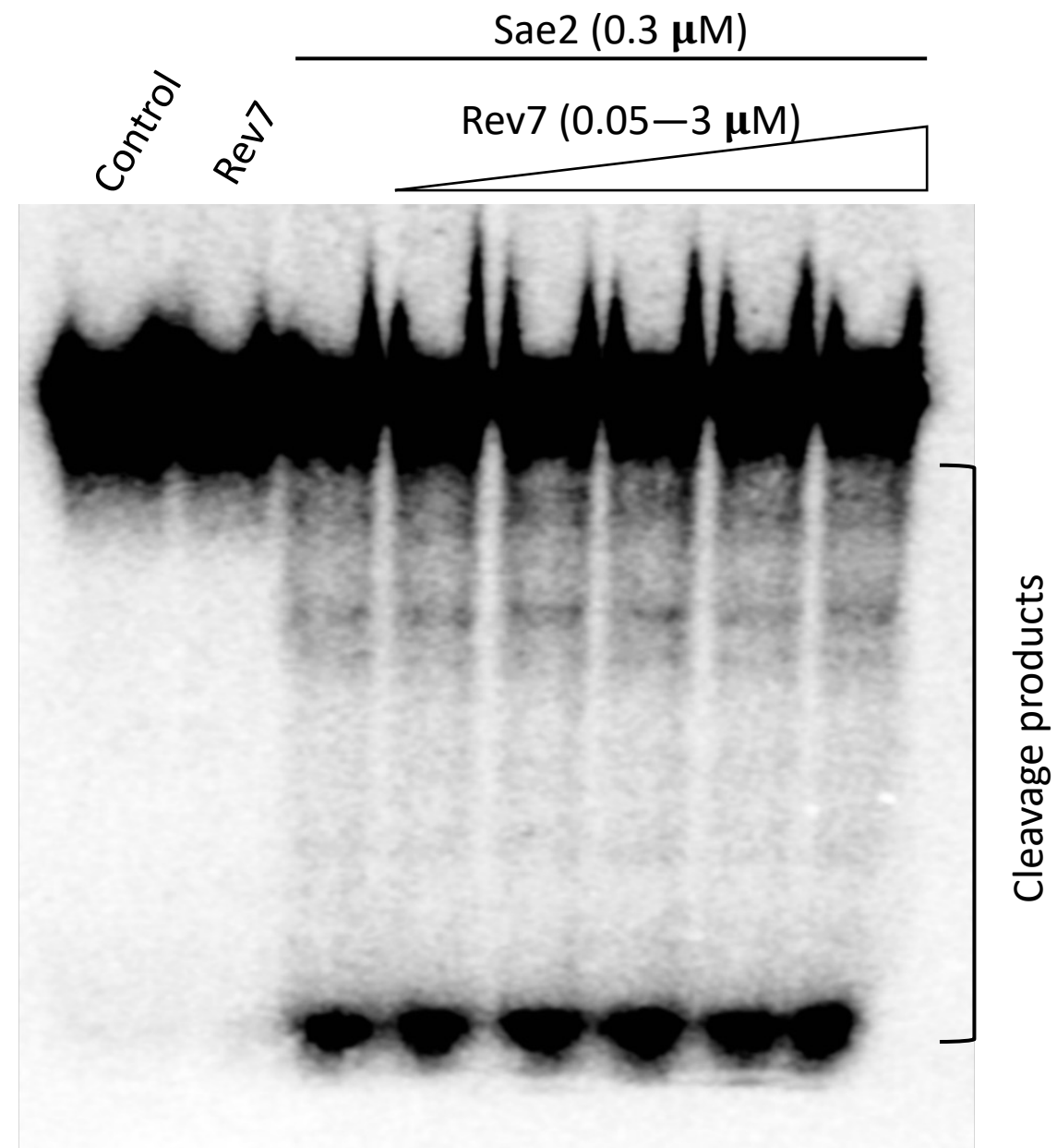

Supplement: Figure 5—figure supplement 1—source data 2. [file elife-96933-fig5-figsupp1-data2.zip › Figure 5-figure supplement 1-source data 2/Figure 5-figure supplement 1-source data 2.pdf]

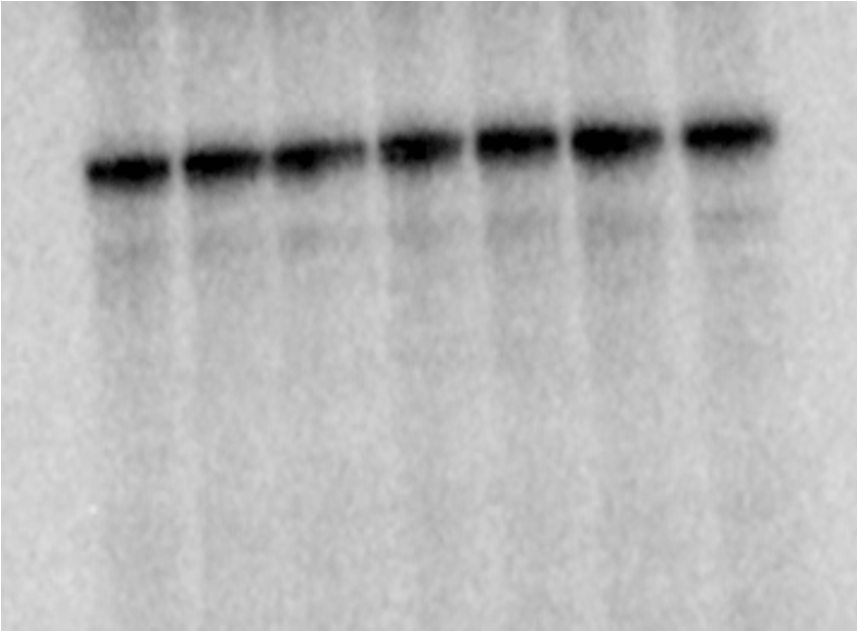

Supplement: Figure 6—source data 1. [file elife-96933-fig6-data1.zip › Figure 6-source data 1/Figure 6-source data 1B.tif]

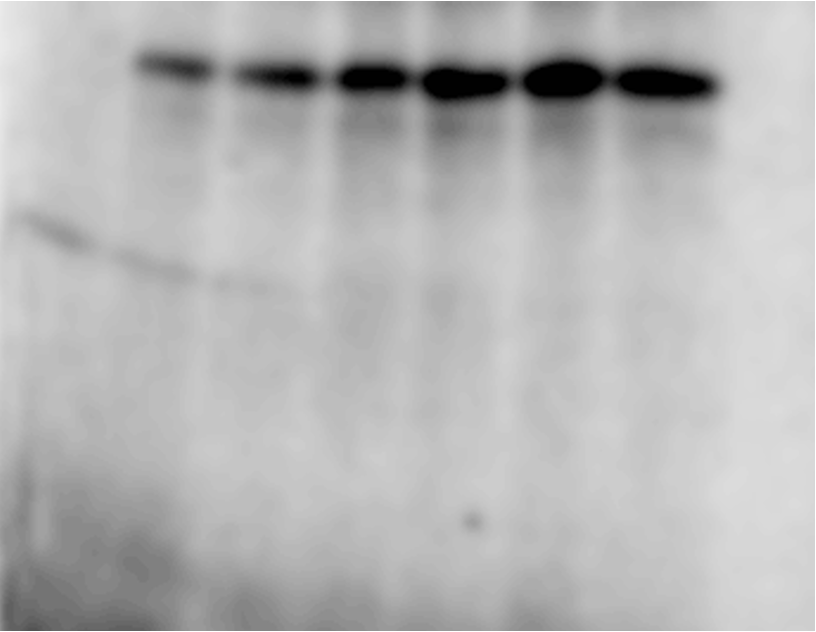

Supplement: Figure 6—source data 1. [file elife-96933-fig6-data1.zip › Figure 6-source data 1/Figure 6-source data 1A.tif]

A.

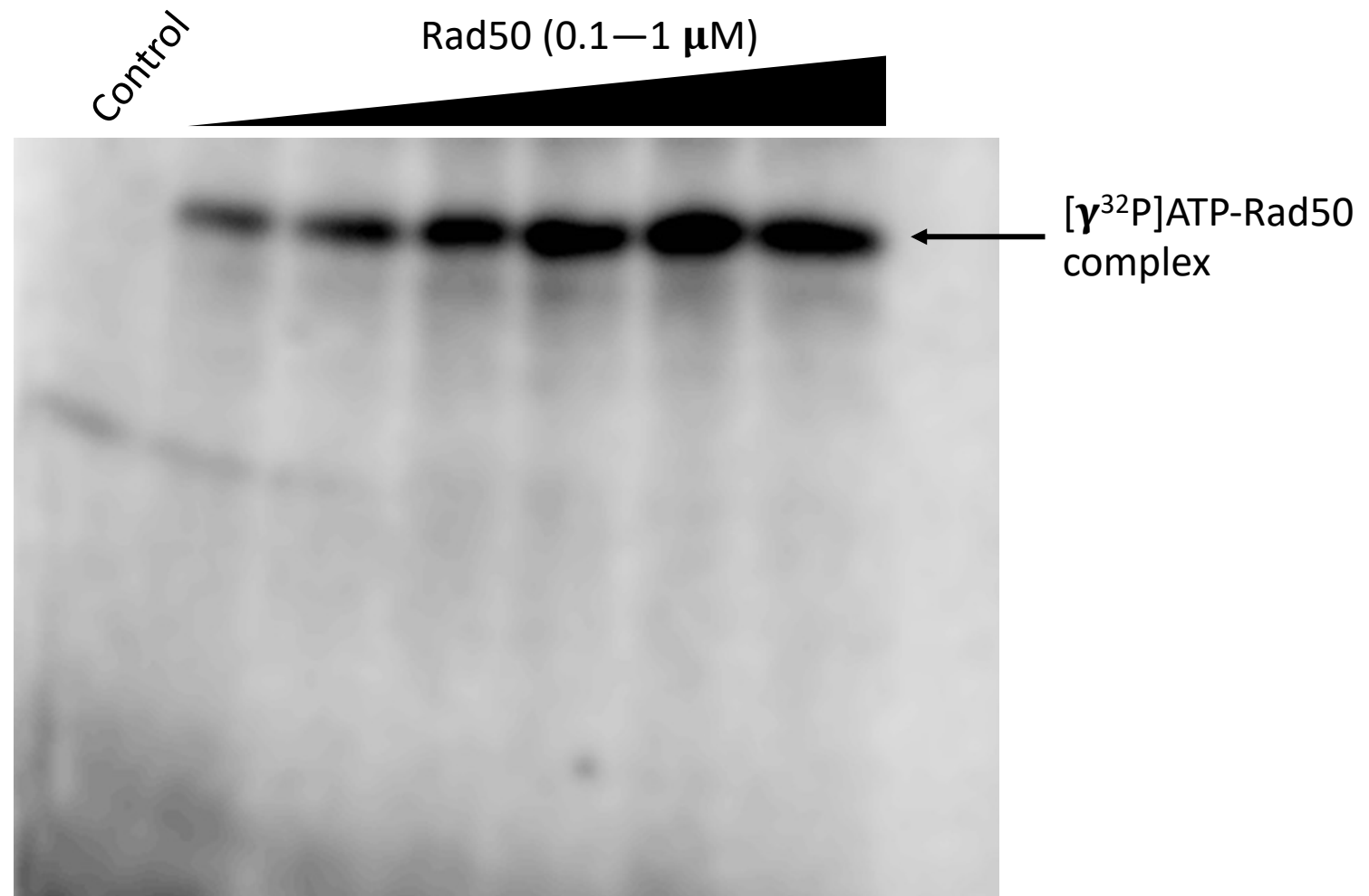

B.

Rad50 (0.2  $\mu\text{M}$ )

Rev7 (0.5—6  $\mu\text{M}$ )

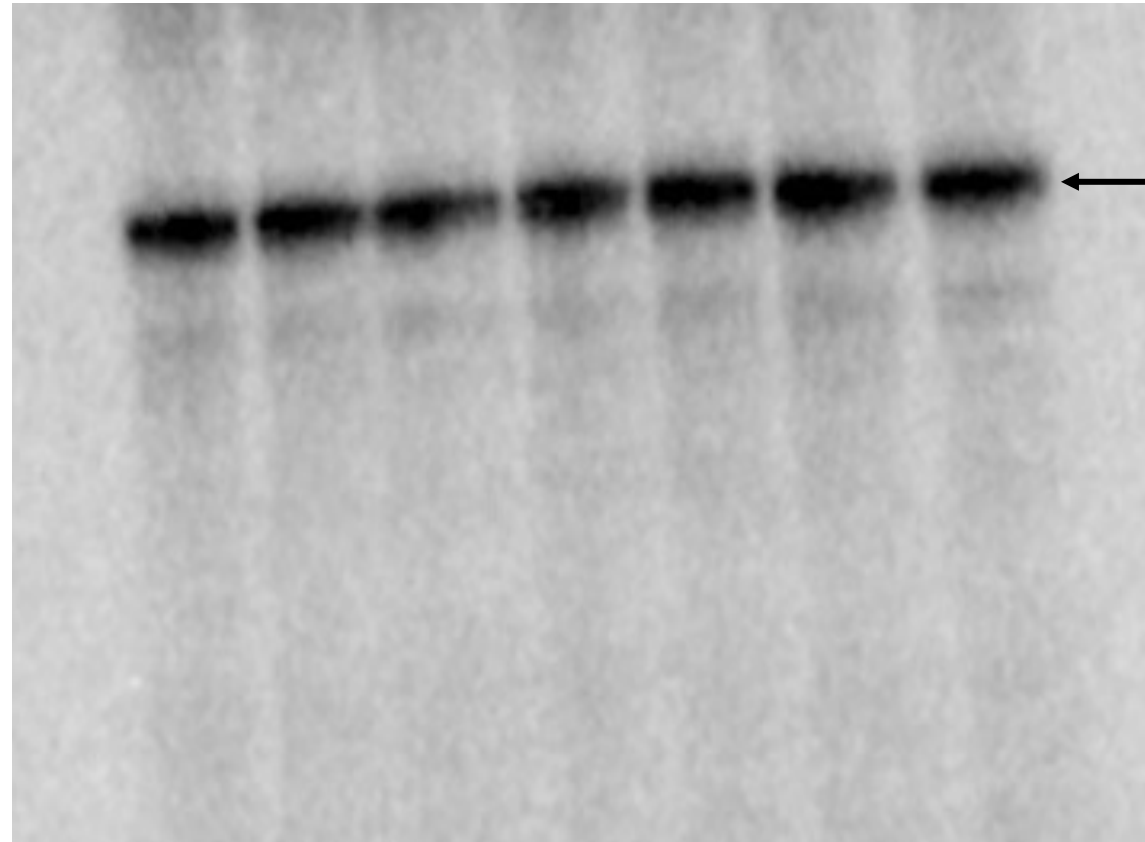

$[\gamma^{32}\text{P}]\text{ATP-Rad50}$   
complex

Supplement: Figure 6—source data 2. [file elife-96933-fig6-data2.zip › Figure 6-source data 2/Figure 6-source data 2.pdf]

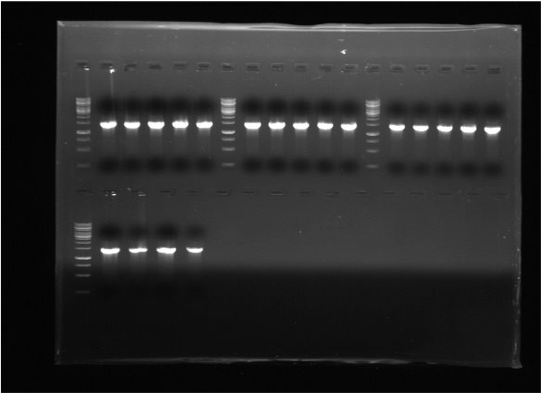

Supplement: Figure 8—source data 2. [file elife-96933-fig8-data2.zip › Figure 8-source data 2/Figure 8-source data 2.tif]

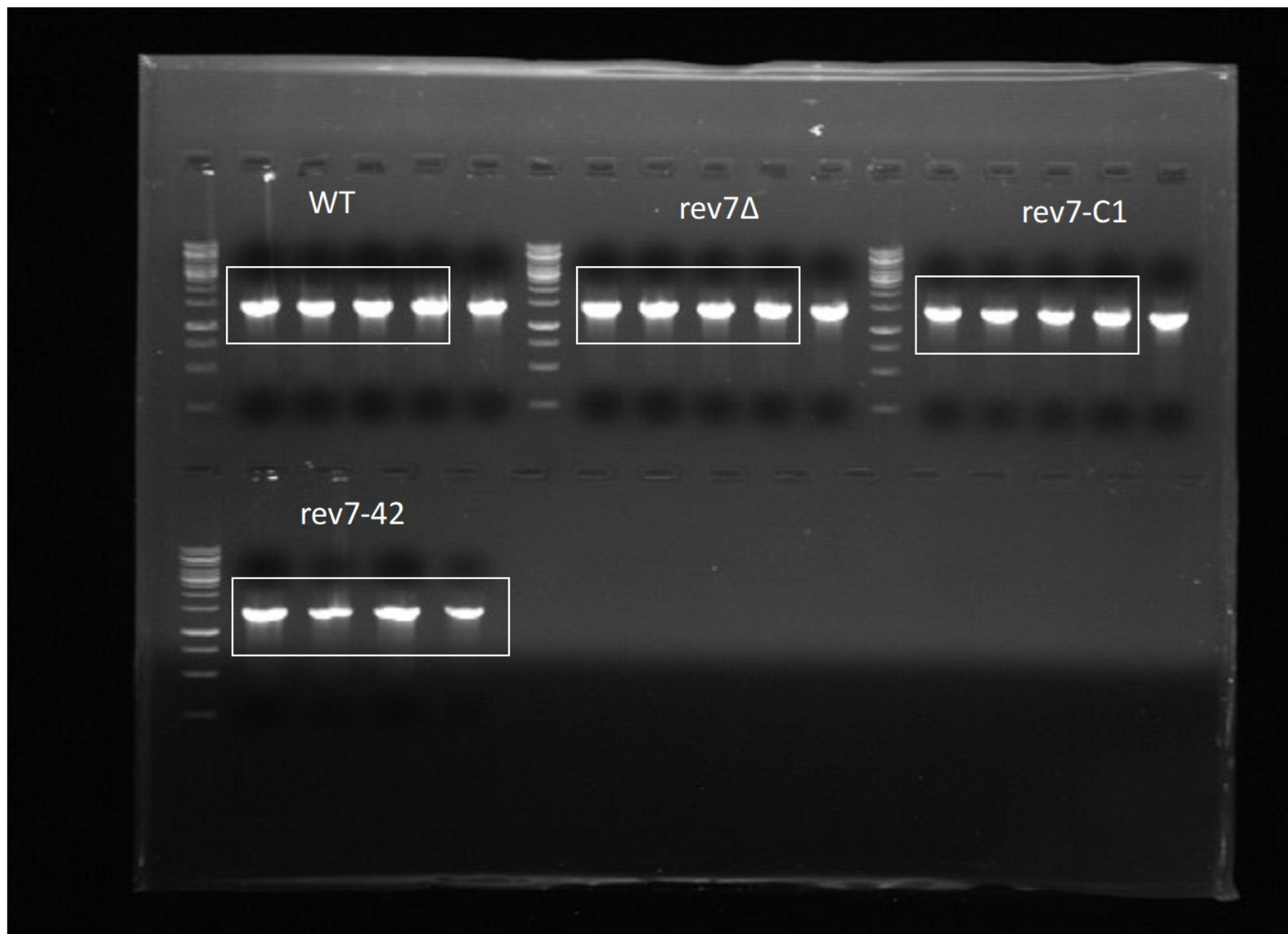

Supplement: Figure 8—source data 2. [file elife-96933-fig8-data2.zip › Figure 8-source data 2/Figure 8-source data 2.pdf]

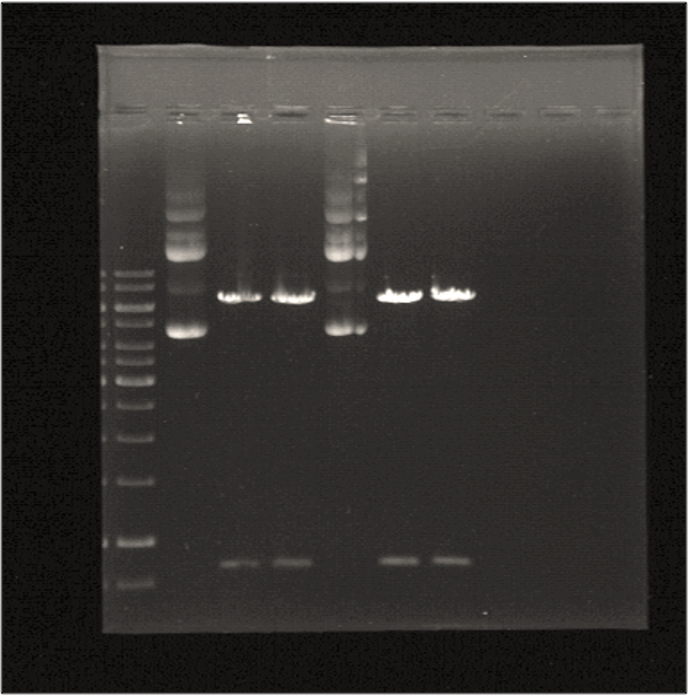

Supplement: Figure 9—figure supplement 2—source data 1. [file elife-96933-fig9-figsupp2-data1.zip › Figure 9-supplement 2-source data 1/Figure 9-figure supplement 2-source data 1A.tif]

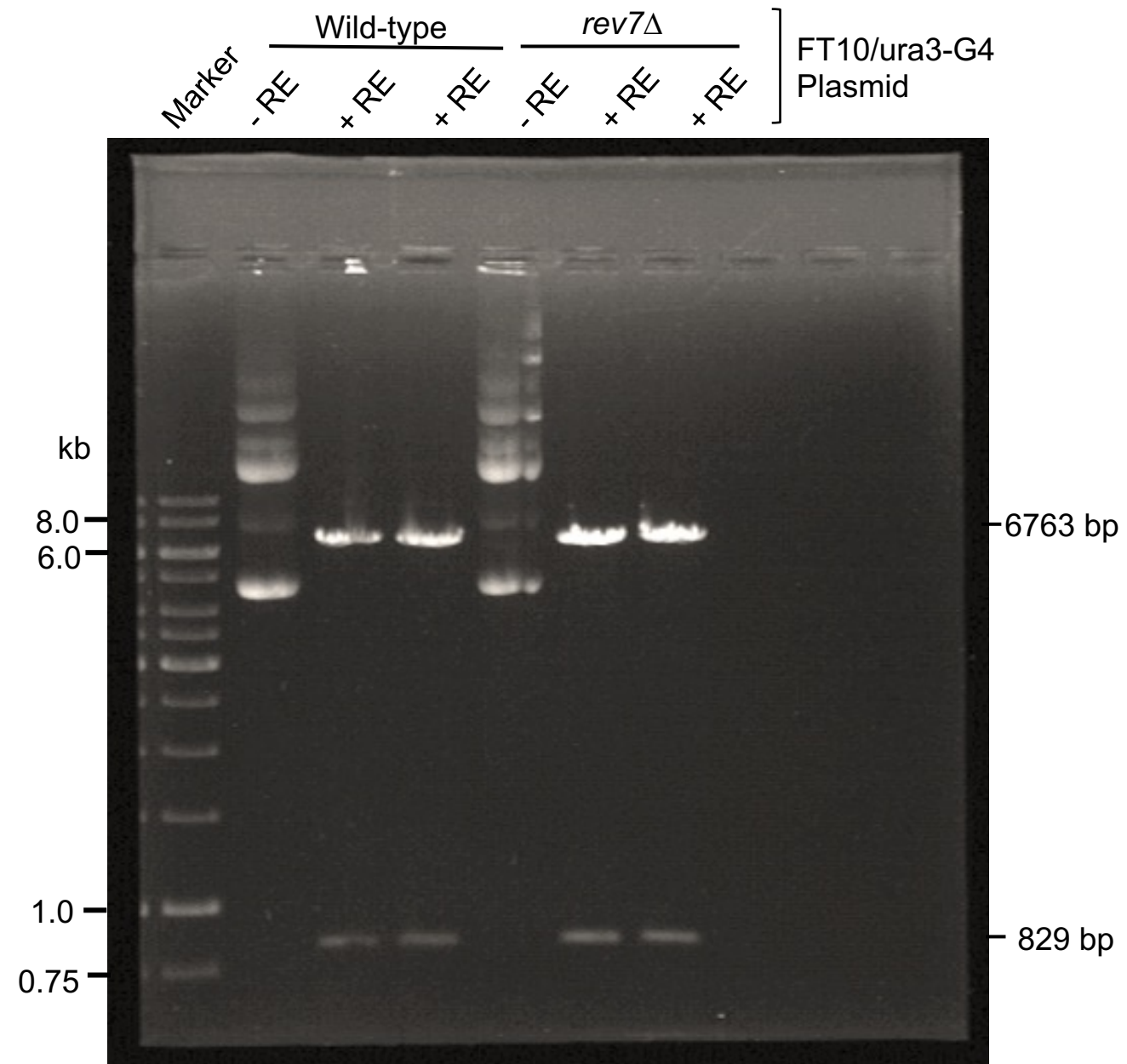

Supplement: Figure 9—figure supplement 2—source data 1. [file elife-96933-fig9-figsupp2-data1.zip › Figure 9-supplement 2-source data 1/Figure 9-figure supplement 2-source data 1B.pdf]
