# Supplementary material for: Saccharomyces cerevisiae Rev7 promotes non-homologous end-joining by blocking Mre11 nuclease and Rad50’s ATPase activities and homologous recombination
Source: eLife. 2024 Dec 4;13:RP96933. doi: 10.7554/eLife.96933 (PMC11616998; doi:10.7554/eLife.96933)
Supplement: Supplementary file 1. — Residues in bold are present in the C-terminal safety-belt region of Rev7 protein. [file elife-96933-supp1.docx]

| Mre11 (position of amino acid residue) | Rev7 (position of amino acid residue) | Distance (Å) | Mre11-residue | Rev7-residue | Mre11-pLDDT | Rev7- pLDDT |
| --- | --- | --- | --- | --- | --- | --- |
| A_0181 | **B_0206** | 0.73 | ARG | **ASP** | 74.44 | 65.25 |
| A_0062 | B_0126 | 1.14 | LYS | MET | 77.81 | 71.25 |
| A_0130 | **B_0242** | 1.27 | GLY | **GLY** | 42.81 | 20.97 |
| A_0134 | **B_0241** | 1.36 | LEU | **PHE** | 72.12 | 22.19 |
| A_0131 | **B_0242** | 1.38 | ASP | **GLY** | 42.41 | 20.97 |
| A_0132 | **B_0241** | 1.86 | SER | **PHE** | 51.47 | 22.19 |
| A_0130 | **B_0243** | 2.16 | GLY | **SER** | 42.81 | 20.88 |
| A_0130 | **B_0241** | 2.23 | GLY | **PHE** | 42.81 | 22.19 |
| A_0131 | **B_0241** | 2.23 | ASP | **PHE** | 42.41 | 22.19 |
| A_0679 | B_0082 | 2.24 | LYS | LYS | 26.53 | 81.81 |
| A_0131 | **B_0240** | 2.52 | ASP | **ILE** | 42.41 | 26.36 |
| A_0130 | B_0130 | 2.74 | GLY | LYS | 42.81 | 72.25 |
| A_0181 | B_0207 | 2.77 | ARG | VAL | 74.44 | 63.84 |
