# Supplementary material for: Saccharomyces cerevisiae Rev7 promotes non-homologous end-joining by blocking Mre11 nuclease and Rad50’s ATPase activities and homologous recombination
Source: eLife. 2024 Dec 4;13:RP96933. doi: 10.7554/eLife.96933 (PMC11616998; doi:10.7554/eLife.96933)
Supplement: Supplementary file 2. — Residues in bold are present in the C-terminal safety-belt region of Rev7 protein. [file elife-96933-supp2.docx]

| Rad50 (position of amino acid residues) | Rev7 (positions of amino acid residues) | Distance (Å) | Rad50-residue | Rev7-residue | Rad50- pLDDT | Rev7- pLDDT |
| --- | --- | --- | --- | --- | --- | --- |
| A_0603 | B_0188 | 1.26 | ARG | ASP | 73.56 | 51.81 |
| A_0582 | B_0156 | 1.45 | LYS | LEU | 73 | 69 |
| A_0599 | B_0084 | 1.88 | HIS | ASN | 73.56 | 68.56 |
| A_0596 | B_0184 | 2.3 | LYS | GLU | 75.75 | 62.81 |
| A_1132 | **B_0245** | 2.5 | LYS | **PHE** | 81 | 18.19 |
| A_0581 | B_0172 | 2.58 | GLN | GLU | 72.12 | 80.44 |
| A_1129 | **B_0245** | 2.6 | VAL | **PHE** | 79 | 18.19 |
| A_0599 | B_0085 | 2.78 | HIS | ASP | 73.56 | 74.62 |
| A_0577 | B_0168 | 2.85 | GLU | LYS | 71.31 | 77.94 |
| A_0603 | B_0189 | 2.85 | ARG | ASN | 73.56 | 46.66 |
