# Supplementary material for: Saccharomyces cerevisiae Rev7 promotes non-homologous end-joining by blocking Mre11 nuclease and Rad50’s ATPase activities and homologous recombination
Source: eLife. 2024 Dec 4;13:RP96933. doi: 10.7554/eLife.96933 (PMC11616998; doi:10.7554/eLife.96933)
Supplement: Supplementary file 3. — The p-values were obtained by comparing the percentage of non-homologous end-joining observed for the indicated single- or double-gene deletions versus either the wild-type (WT) or rev7Δ strain, using non-parametric one-way ANOVA Dunnett test. [file elife-96933-supp3.docx]

| **Reference strain** | **Strain** | **p-values** |
| --- | --- | --- |
| **WT** | *rev1Δ* | 0.0461 |
|  | *rev3Δ* | 0.0011 |
|  | *rev7Δ* | <0.0001 |
|  | *mre11Δ* | <0.0001 |
|  | *sae2Δ* | 0.7111 |
|  | *rev1Δ rev3Δ* | <0.0001 |
|  | *rev1Δ rev7Δ* | <0.0001 |
|  | *rev3Δ rev7Δ* | <0.0001 |
|  | *mre11Δ rev7Δ* | <0.0001 |
|  | *sae2Δ rev7Δ* | <0.0001 |
|  | *rev7-C1* | <0.0001 |
|  | *rev7-42* | 0.9910 |
| ***rev7𝛥*** | *rev1Δ rev7Δ* | 0.2731 |
|  | *rev3Δ rev7Δ* | 0.9958 |
|  | *mre11Δ rev7Δ* | 0.0029 |
|  | *sae2Δ rev7Δ* | 0.2220 |
|  | *rev7-C1* | 0.0022 |
|  | *rev7-42* | <0.0001 |
