# Supplementary material for: Saccharomyces cerevisiae Rev7 promotes non-homologous end-joining by blocking Mre11 nuclease and Rad50’s ATPase activities and homologous recombination
Source: eLife. 2024 Dec 4;13:RP96933. doi: 10.7554/eLife.96933 (PMC11616998; doi:10.7554/eLife.96933)
Supplement: Supplementary file 5. [file elife-96933-supp5.docx]

| **Strain** | **Genotype** | **Source** |
| --- | --- | --- |
| W1588-4C | *MATa ura3-1 trp1-1 leu2-3 112 his3-11 15 ade2-1 can1-100 ybp1-1 RAD5^+^* | Paeschke *et al*., 2011 |
| KP013 | *MATa ura3-1 trp1-1 leu2-3 112 his3-11 15 ade2-1 can1-100 ybp1-1 RAD5^+^ bar1Δ::HIS3* | Paeschke *et al*., 2011 |
| KMY017 | *MATa ura3-1 trp1-1 leu2-3 112 his3-11 15 ade2-1 can1-100 ybp1-1 RAD5^+^ bar1Δ::HIS3 rev7Δ:: kanMX4* | This Study |
| KMY042 | *MATa ura3-1 trp1-1 leu2-3 112 his3-11 15 ade2-1 can1-100 ybp1-1 RAD5^+^ bar1Δ::HIS3 rev1Δ:: hphNT1* | This Study |
| KMY061 | *MATa ura3-1 trp1-1 leu2-3 112 his3-11 15 ade2-1 can1-100 ybp1-1 RAD5^+^ bar1Δ::HIS3 rev3Δ::KanMX4* | This Study |
| KMY018 | *MATa ura3-1 trp1-1 leu2-3 112 his3-11 15 ade2-1 can1-100ybp1-1 RAD5^+^ bar1Δ::HIS3 mre11Δ:: hphNT1* | This Study |
| KMY141 | *MATa ura3-1 trp1-1 leu2-3 112 his3-11 15 ade2-1 can1-100 ybp1-1 RAD5^+^ bar1Δ::HIS3 rev7Δ::rev7-C1 (203-245 aaΔ)-9MYC-hphNT1* | This Study |
| KMY143 | *MATa ura3-1 trp1-1 leu2-3 112 his3-11 15 ade2-1 can1-100 ybp1-1 RAD5^+^ bar1Δ::HIS3 rev7Δ::rev7-42 (1-203 aaΔ)-3MYC-KANMX4* | This Study |
| LSY1375 | *MATa ura3-1 trp1-1 leu2-3 112 his3-11 15 ade2-1 can1-100 ybp1-1 RAD5^+^ mre11-D56N, H125N* | Krogh *et al*., 2005 |
| LSY1091 | *MATa ura3-1 trp1-1 leu2-3 112 his3-11 15 ade2-1 can1-100 ybp1-1 RAD5^+^ sae2Δ:: kanMX6* | Krogh *et al*., 2005 |
| KMY058 | *MATa ura3-1 trp1-1 leu2-3 112 his3-11 15 ade2-1 can1-100 ybp1-1 RAD5^+^ bar1Δ::HIS3 rev1Δ:: hphNT1* *rev3Δ∷KanMX4* | This Study |
| KMY059 | *MATa ura3-1 trp1-1 leu2-3 112 his3-11 15 ade2-1 can1-100 ybp1-1 RAD5^+^ bar1Δ::HIS3 rev7Δ:: kanMX4 rev3Δ:: hphNT1* | This Study |
| KMY048 | *MATa ura3-1 trp1-1 leu2-3 112 his3-11 15 ade2-1 can1-100 ybp1-1 RAD5^+^ bar1Δ::HIS3 rev1Δ:: hphNT1 rev7Δ:: kanMX4* | This Study |
| KMY050 | *MATa ura3-1 trp1-1 leu2-3 112 his3-11 15 ade2-1 can1-100 ybp1-1 RAD5^+^ rev7Δ:: kanMX4 mre11Δ:: hphNT1* | This Study |
| KMY053 | *MATa ura3-1 trp1-1 leu2-3 112 his3-11 15 ade2-1 can1-100 ybp1-1 RAD5^+^ mre11-D56N, H125N rev7Δ:: kanMX4* | This Study |
| KMY060 | *MATa ura3-1 trp1-1 leu2-3 112 his3-11 15 ade2-1 can1-100 ybp1-1 RAD5^+^ sae2Δ:: kanMX6* *rev7Δ:: hphNT1* | This Study |
| KP038 | *MATa ura3–52 lys2–801 ade2–101 trp1-Δ63 his1-Δ200 leu2-Δ1 pif1-m2* | Paeschke *et al*., 2011 |
| YW714 | *MATα ade2::SD2-::URA3 his31 leu20 ura30* | Karathanasis and Wilson, 2002 |
| KMY136 | *MATα ade2::SD2-::URA3 his31 leu20 ura30 rev7Δ::hphNT1* | This Study |
| KMY137 | *MATα ade2::SD2-::URA3 his31 leu20 ura30 mre11Δ::hphNT1* | This Study |
| KMY144 | *MATα ade2::SD2-::URA3 his31 leu20 ura30 rev1Δ::hphNT1* | This Study |
| KMY145 | *MATα ade2::SD2-::URA3 his31 leu20 ura30 rev3Δ::hphNT1* | This Study |
| KMY146 | *MATα ade2::SD2-::URA3 his31 leu20 ura30  rev7Δ::REV7-C1 (203-245 aaΔ)-9MYC-hphNT1* | This Study |
| KMY147 | *MATα ade2::SD2-::URA3 his31 leu20. ura30  rev7Δ::REV7-42 (1-203 aaΔ)-3MYC-KANMX4* | This Study |
| PJ69-4A | *MATa trpl-901 leu2-3,112 ura3-52 his3-200 ga14∆ ga180∆ LYS2::GALl-HIS3 GAL2-ADE2 met2::GAL7-lacZ* | James *et al*., 1996 |
| KMY023 | *MATa trpl-901 leu2-3,112 ura3-52 his3-200 ga14∆ ga180∆ LYS2::GALl-HIS3 GAL2-ADE2 met2::GAL7-lacZ  rev3Δ:: hphNT1* | This Study |
| KMY139 | *MATa trpl-901 leu2-3,112 ura3-52 his3-200 ga14∆ ga180∆ LYS2::GALl-HIS3 GAL2-ADE2 met2::GAL7-lacZ mre11∆::KANMX4 rad50∆::URA3 xrs2∆::HphNT1* | This Study |
| BJ5464 | MATα ura3-52 trp1 leu2-Δ1 his3-Δ200 pep4::HIS3 prb1-Δ1.6R can1 GAL | Johnson *et al*., 2006 |
| LSY2172-24C | MATa rad51::LEU2 ade3::GAL1-HO | Mimitou and Symington, 2010 |
| LSY2265-10D | MATa rad51::LEU2 mre11-H125N::URA3::mre11-H125N ade3::GAL1-HO | Mimitou and Symington, 2010 |
| KMY170 | MATa rad51::LEU2 ade3::GAL1-HO rev7*Δ*:: *kanMX4* | This study |
| KMY171 | MATa rad51::LEU2 mre11-H125N::URA3::mre11-H125N ade3::GAL1-HO rev7*Δ*:: *kanMX4* | This study |
