# Supplementary material for: Saccharomyces cerevisiae Rev7 promotes non-homologous end-joining by blocking Mre11 nuclease and Rad50’s ATPase activities and homologous recombination
Source: eLife. 2024 Dec 4;13:RP96933. doi: 10.7554/eLife.96933 (PMC11616998; doi:10.7554/eLife.96933)
Supplement: Supplementary file 6. — The bold letters correspond to restrictions sites. [file elife-96933-supp6.docx]

| **Primer names** | **Sequence** **(5'—3')** | **Plasmid DNA/primers** |
| --- | --- | --- |
| OSB01-FP | GC**GGATCC**ATGAATAGATGGGTAGAGAAGTG | pET28a_ScREV7,  pET28a_ScREV7-C1 |
| OSB02-RP | GC**AAGCTT**TTCTTCAAATTTCATTTTTGCAC | pET28a_ScREV7 |
| OSB03-FP | **CATATG**ATGAATAGATGGGTAGAGAAGTG | pGBKT7_ScREV7,  pGADT7_ScREV7 |
| OSB04-RP | **GGATCC**TTCTTCAAATTTCATTTTTGCAC | pGBKT7_ScREV7,  pGADT7_ScREV7 |
| OSB05-FP | ATGCCT**CATATG**ATGGACTATCCTGATCCAGACACACAATAAGG | pGADT7_ScMRE11 |
| MRE11_RP | ATGCCT**GAATTC**CTATTTTCTTTTCTTAGGAAGGAGGACTTCC | pGADT7_ScMRE11 |
| OSB11-FP | ATCCAAGAAGAAAAAAAAAATAGTAATCGTTGCGTCAGCTTTATGCGTACGCTGCAGGTCGAC | REV7 Knockout |
| OSB12-RP | ACATTTAATTTTAATTCCATTCTTCAAATTTCATTTTTGCACTTAATCGATGAATTCGAGCTCG | REV7 Knockout |
| OSB13-FP | ATATGCATATACGTCTACACTAC | REV7 Knockout |
| OSB14-RP | CTGCAGCGAGGAGCCGTAAT (KANB) | REV7 Knockout |
| OSB33-FP | CGC**GGATCC**ATGAGCGCTATCTATAAATTATC | pESUMO_ScRAD50 |
| OSB34-RP | CCG**CTCGAG**TCAATAAGTGACTCTGTTAATATC | pESUMO_ScRAD50             & pGADT7_ScRAD50 |
| OSB35-FP | CCG**GAATTC**ATGAGCGCTATCTATAAATTATC | pGADT7_ScRAD50 |
| OSB36-FP | GGAATTC**CATATG**ATGTGGGTAGTACGATACCAG | pGADT7_ScXRS2 |
| OSB37-RP | CG**GGATCC**TTATCCTTTTCTTCTTTTGAA | pGADT7_ScXRS2 |
| OSB52-FP | ATTGACGCAAGTTGTACCTGCTCAGATCCGATAAAACTCGACTATGCGTACGCTGCAGGTCGAC | MRE11 Knockout |
| OSB53-RP | TGGTTATAAATAGGATATAATATAATATAGGGATCAAGTACAACTAATCGATGAATTCGAGCTCG | MRE11 Knockout |
| OSB54-FP | AAGGCATCTACAAATCTCATTG | MRE11 Knockout |
| OSB133-FP | AGGCAAAAATCACAAATTGAGTGGGTCGATATTAACAGAGTCACTTATCGTACGCTGCAGGTCGAC | RAD50 Knockout |
| OSB075-RP | TAATTAATCAATCAAAGTCTATCCCTTCGTAGATATTATGGGGTCTTTATCGATGAATTCGAGCTCG | RAD50 Knockout |
| OSB076-RP | TCCTTGGTTAACAACGGTG | RAD50 Knockout |
| OSB134-FP | ACGACGACGATGACGACGGTCCGAAGTTTACGTTCAAAAGAAGAAAAGGACGTACGCTGCAGGTCGAC | XRS2 Knockout |
| OSB078-RP | TGCAAAATATAATTTAATGAAATTGGAAATACTCGGAAAATTTATCATTAATCGATGAATTCGAGCTCG | XRS2 Knockout |
| OSB079-RP | TTGGAGTATTCAAAGAGGCTAC | XRS2 Knockout |
|  |  |  |
| OSB55-FP | TTCTCAAAATAAATCGATACTGCATTTCTAGGCATATCCAGCGATGCGTACGCTGCAGGTCGAC | REV1 Knockout |
| OSB56-FP | AACTGCGTGTTTACTGTATGCTGAAATGTTTTTTTTTTTTTAATTCAATCGATGAATTCGAGCTCG | REV1 Knockout |
| OSB57-FP | TTTACACAGACCAAGACGG | REV1 Knockout |
| OSB58-FP | TCAATACAAAACTACAAGTTGTGGCGAAATAAAATGTTTGGAAATGCGTACGCTGCAGGTCGAC | REV3 Knockout |
| OSB59-RP | ACAAATAACTACTCATCATTTTGCGAGACATATCTGTGTCTAGATTAATCGATGAATTCGAGCTCG | REV3 Knockout |
| OSB60-FP | ATCCCTGTGGTCTCCTACC | REV3 Knockout |
| OSB116-FP | ATCCAAGAAGAAAAAAAAAATAGTAATCGTTGCGTCAGCTTTATGAATAGATGGGTAGAGAAGTG | *rev7-c1* truncation  (rev7-c1-9MYC-hphNT1) |
| OSB117-RP | GTCGACCTGCAGCGTACGGACTAAAGAAGTGAGTTTTATTTTAGG | *rev7-c1* truncation  (rev7-c1-9MYC-hphNT1) |
| OSB118-FP | CCTAAAATAAAACTCACTTCTTTAGTCCGTACGCTGCAGGTCGAC | *rev7-c1* truncation  (rev7-c1-9MYC-hphNT1) |
| OSB070-RP | TACTTAGAGACATTTAATTTTAATTCCATTCTTCAAATTTCATTTTTGCACTTAATCGATGAATTCGAGCTCG | *rev7-c1* truncation  (rev7-c1-9MYC-hphNT1) |
| OSB122-FP | ATCCAAGAAGAAAAAAAAAATAGTAATCGTTGCGTCAGCTTTATGGGTTCTGACGTGGGGCC | *rev7-42 aa* truncation  (rev7-42-3MYC-KANMX) |
| OSB120-RP | GTCGACCTGCAGCGTACGAAACAAAGATCCAAAAATGCTC | *rev7-42 aa* truncation  (rev7-42-3MYC-KANMX) |
| OSB121-FP | GAGCATTTTTGGATCTTTGTTTCGTACGCTGCAGGTCGAC | *rev7-42 aa* truncation  (rev7-42-3MYC-KANMX) |
| OSB61-FP | GGAATTC**CATATG**TCATTCACCTTGCCGCAGTTC | pGBKT7_REV7-N1 |
| OSB62-FP | GGAATTC**CATATG**ACCCACGTTTACAGATTTTCC | pGBKT7_REV7-N2 |
| OSB63-FP | GGAATTC**CATATG**TTGGAACTAGGACATAAGTTGG | pGBKT7_REV7-N3 |
| OSB64-RP | CG**GGATCC**TTAGACTAAAGAAGTGAGTTTTATTTTAGG | pGBKT7_REV7-C1 |
| OSB65-RP | CG**GGATCC**TTATTCTGCTTTTTCCTCCAAACTATC | pGBKT7_REV7-C2 |
| OSB66-RP | CG**GGATCC**TTACAATTCGATCGCATTAATAACTGC | pGBKT7_REV7-C3 |
| OSB80-FP | CG**GGATCC**ATGTCGAAAGCTACATATAAG | pFAT10_ura3-G4 |
| OSB81-RP | **CCC**GGT**CCCCCC**AACGACAACA**CCC**AATCAACCAATCGTAACCTTC | pFAT10_ura3-G4 |
| OSB82-FP | **GGG**TGTTGTCGTT**GGGGGG**ACC**GGG**ATGACACCCGGTGTGGG | pFAT10_ura3-G4 |
| OSB83-RP | AC**ATGCAT**GCTTAGTTTTGCTGGCCGCATC | pFAT10_ura3-G4 |
| OSB129-RP | CTCATAGCATCCAACGACAACACGCAATCAACCAATCGTAACCTTC | pFAT10_ura3-G4 mutant |
| OSB130-FP | GCGTGTTGTCGTTGGATGCTATGAGATGACACCCGGTGTGGG | pFAT10_ura3-G4 mutant |
| OSB125-RP | GC**AAGCTT**TTAGACTAAAGAAGTGAGTTTTATTTTAGG | pET28_ScRev7-C1 |
| REV7GFP-FP | GC**TCTAGA**ATGAATAGATGGGTAGAGAAG | pPROEX_ScREV7-eGFP |
| REV7GFP-RP | CCG**CTCGAG**GTTAATTAACCCGGGGATCCGAAACAAAGATCCAAAAATGC | pPROEX_ScREV7-eGFP |
| OSB150-FP | ATGCCT**CATATG**ATGGTGACTGGTGAAG | pGADT7-SAE2 |
| OSB151-RP | ATGCCT**GAATTC**TTAACATCTTAGCATATA | pGADT7-SAE2 |
| OSB152-RP | CCG**CTCGAG**GTTAATTAACCCGGGGATCCGGACTAAAGAAGTGAGTTTTATTTTAGG | pPROEX_ScREV7C1-eGFP |
