# Supplementary material for: Saccharomyces cerevisiae Rev7 promotes non-homologous end-joining by blocking Mre11 nuclease and Rad50’s ATPase activities and homologous recombination
Source: eLife. 2024 Dec 4;13:RP96933. doi: 10.7554/eLife.96933 (PMC11616998; doi:10.7554/eLife.96933)
Supplement: Supplementary file 7. [file elife-96933-supp7.docx]

| **Oligonucleotide name** | **Sequence** **(5'—3')** |
| --- | --- |
| OSB 17  Duplex-41 bp | GCCGTGATCACCAATGCAGATTGACGAACCTTTGCCCACGT |
| OSB 20  Duplex-41 bp | ACGTGGGCAAAGGTTCGTCAATCTGCATTGGTGATCACGGC |
| OSB 41  Duplex-60 bp | GGGTGAACCTGCAGGTGGGCAAAGATGTCCTAGCAATGTAATCGTCAAGCTTTATGCCGT |
| OSB 42  Duplex-60 bp | ACGGCATAAAGCTTGACGATTACATTGCTAGGACATCTTTGCCCACCTG CAGGTTCACCC |
| OSB28 (4G3)  G4-DNA | AATTCT**GGG**TGTGT**GGG**TGTGT**GGG**TGTGT**GGG**TGTGG |
| OSB 92 (TP)      G4-DNA | TGGACCAGACCTAGCAGCTAT**GGGGG**AGCT**GGGG**AAGGT**GGG**AATGTGA |
| ODN 6G3       G4-DNA | AATTCT**GGG**TGTGT**GGG**TGTGT**GGG**TGTGT**GGG**TGTGT**GGG**TGTGT**GGG**TGTGG |
| OSB132  (TP-G4mutant) | TGGACCAGACCTAGCACTATCTGCAAGTCAAGTTGACTACGTATACATA |
