# Supplementary material for: Saccharomyces cerevisiae Rev7 promotes non-homologous end-joining by blocking Mre11 nuclease and Rad50’s ATPase activities and homologous recombination
Source: eLife. 2024 Dec 4;13:RP96933. doi: 10.7554/eLife.96933 (PMC11616998; doi:10.7554/eLife.96933)
Supplement: Supplementary file 8. [file elife-96933-supp8.docx]

| **Primer** | **Sequence (5'—3')** |
| --- | --- |
| 0.7 kb (Forward) | TTTAGGATACTTTCACGCTTTA |
| 0.7 kb (Reverse) | TTAGCTTGTACCAGAGGAA |
| 3 kb (Forward) | GTCGTTCATATCTAAAGGAGTTAT |
| 3 kb (Reverse) | CCTACCGCACCTTCTAAG |
| PRE1 (Forward) | AAATCTTTACGGTGGCAAA |
| PRE1 (Reverse) | CGCTAGAATGACAGAATCC |
| HO efficiency (Forward) | TCAATGATTAAAATAGCATAGTCGGGT |
| HO efficiency (Reverse) | CGTCAACCACTCTACAAAACCA |
